# Development of a Multimodal Model Combining Imaging and Clinical Data to Predict Bone Cement Leakage During Vertebroplasty for Fresh Thoracolumbar Compression Fractures

**DOI:** 10.1002/jsp2.70227

**Published:** 2026-11-08

**Authors:** Wangim Choi, Jianan Chen, Jincheng Qiu, Tianyu Xie, Yong Li, Zhiwei Pan, Zaoqiang Zhang, Guoxi Huang, Nianchun Liao, Youxi Lin, Zhihuai Deng, Xianjian Qiu, Bo Gao, Dongsheng Huang, Hang Zhou, Zizhao Wu, Wenjie Gao

**Affiliations:** ^1^ Department of Orthopedics Sun Yat‐Sen Memorial Hospital Guangzhou China; ^2^ Department of Orthopedics The Third Affiliated Hospital of Sun Yat‐Sen University Guangzhou China; ^3^ Department of Minimally Invasive Spine Surgery Panyu Hospital of Chinese Medicine Guangzhou Guangdong Province China; ^4^ Shenshan Medical Center Sun Yat‐Sen Memorial Hospital, Sun Yat‐sen University Shanwei Guangdong P. R. China; ^5^ Department of Radiology Sun Yat‐Sen Memorial Hospital Guangzhou China; ^6^ Guangzhou University of Chinese Medicine Guangzhou China

**Keywords:** artificial intelligence, bone cement leakage, multimodal model, percutaneous vertebroplasty, radiomics

## Abstract

**Background:**

Bone cement leakage is a common complication of percutaneous vertebroplasty (PVP), but perioperative risk stratification remains challenging. Existing models based on single‐source clinical or imaging features have limited performance. This study aimed to develop and validate multimodal artificial intelligence (AI) models integrating clinical, imaging, and radiomics features to predict bone cement leakage during PVP.

**Methods:**

This multicenter study included 486 treated vertebrae from 378 patients at the North, South, and Shenshan campuses of our hospital. The treated vertebra was the prediction unit, while all vertebrae from the same patient were retained in the same dataset partition and cross‐validation fold. Clinical variables, conventional CT imaging features, and CT‐based radiomics features were extracted. Logistic regression identified independent risk factors. Six machine‐learning algorithms were used to construct clinical, imaging, radiomics, and multimodal models. Performance was evaluated using the area under the receiver operating characteristic curve (AUC), accuracy, F1 score, and decision curve analysis. Independent external and prospective validations were performed, and SHapley Additive exPlanations (SHAP) were used for model interpretation.

**Results:**

Among the 486 treated vertebrae, 215 (44.2%) had cement leakage. Higher body mass index (BMI), greater cement injection volume, cortical destruction, and posterior wall involvement were independently associated with leakage. Multimodal models generally achieved higher AUCs than the corresponding single‐modality models, with AUCs of 0.902–0.939 in the internal test cohort, 0.722–0.929 in the external validation cohort, and 0.834–0.925 in the prospective validation cohort. Patient‐level aggregation and one‐vertebra‐per‐patient sensitivity analyses yielded broadly comparable discrimination. SHAP identified cortical destruction, cement injection volume, and posterior wall involvement as the most influential predictors.

**Conclusions:**

The multimodal models demonstrated good and generally consistent discrimination across the internal test, external validation, and prospective validation cohorts and may support perioperative risk assessment and individualized cement injection planning during PVP.

## 1 Introduction

The spine plays a critical biomechanical role in load bearing and spinal stability. With population aging, osteoporotic vertebral compression fractures (OVCFs) have become increasingly prevalent [1, 2], particularly in the thoracolumbar region, where reduced bone density predisposes the spine to low‐energy trauma. OVCFs are a major cause of pain, functional limitation, and impaired quality of life [3, 4], and acute fractures often require timely intervention [5, 6].

Percutaneous vertebroplasty (PVP) and kyphoplasty (PKP) are widely used minimally invasive treatments that effectively relieve pain and restore vertebral stability [7]. However, bone cement leakage remains a common complication, with reported incidence ranging from 5% to 80% [8, 9, 10], and may result in serious adverse events such as neurological injury and pulmonary embolism [11]. Current perioperative risk assessment relies primarily on fracture morphology, cortical integrity, and bone mineral density, which are often subjective and insufficient for accurate individualized risk prediction.

Recent advances in artificial intelligence (AI), including machine learning (ML), deep learning (DL), and radiomics, enable quantitative analysis of CT images [12]. DL can automatically extract high‐dimensional imaging features, while radiomics captures tissue heterogeneity at the microstructural level. When combined with clinical variables in multimodal frameworks and implemented with algorithms such as random forests and XGBoost, these approaches have shown promise in predicting cement leakage [6, 13, 14, 15, 16]. However, most studies are limited by single‐center designs, suboptimal multimodal integration, limited interpretability, and lack of external validation.

Therefore, this study aimed to develop and validate a multimodal AI model integrating preoperative CT‐based imaging and radiomics features with perioperative clinical data to predict postoperative cement leakage in patients with fresh thoracolumbar OVCFs, with the goal of improving perioperative risk assessment and supporting individualized surgical decision‐making.

## 2 Materials and Methods

### 2.1 Study Design and Participants

This retrospective study was approved by the institutional ethics committee (approval No. SYSKY‐2025‐832‐01), and the requirement for informed consent was waived.

Analyses were conducted at the vertebral level because cement leakage was assessed separately for each treated vertebra. To account for within‐patient correlation, all vertebrae from the same patient were assigned to the same dataset partition and cross‐validation fold. The development cohort included 486 treated vertebrae from 378 patients treated between January 2018 and June 2025 at the North, South, and Shenshan campuses of our hospital. The training cohort comprised 385 vertebrae from 301 patients, and the internal test cohort comprised 101 vertebrae from 77 patients. The distribution of treated vertebrae per patient is provided in Table S1. No patient overlap was identified between the training and internal test cohorts. The external and prospective validation cohorts included 50 vertebrae from 45 patients and 60 vertebrae from 50 patients, respectively, with no patient overlap across cohorts. Both validation cohorts were evaluated without further partitioning. An anonymized patient identifier was retained for each record to support patient‐cluster bootstrap resampling and patient‐level sensitivity analyses.

*Inclusion criteria were*: (1) fresh thoracolumbar vertebral compression fractures confirmed by clinical presentation and imaging; (2) availability of preoperative thoracolumbar CT and MRI demonstrating bone marrow edema, followed by treatment with PVP; (3) osteoporosis confirmed by bone mineral density (BMD) testing; and (4) complete clinical and imaging data.

*Exclusion criteria were*: (1) age < 55 years; (2) spinal cord, cauda equina, or nerve root injury; (3) MRI evidence of old or malignant vertebral compression fractures; and (4) incomplete clinical data or inadequate imaging quality.

### 2.2 Outcome Labeling and Vertebral Identification

Target vertebrae for surgical intervention were identified on preoperative CT and MRI, and postoperative radiographs and CT images were evaluated to determine the presence of bone cement leakage by three spine surgeons (> 5 years of experience) and two senior musculoskeletal radiologists. Bone cement leakage was defined as extravasation of cement beyond the vertebral body. The primary outcome was the presence or absence of cement leakage at each treated vertebra on postoperative imaging. Leakage was further categorized as intervertebral disc, anterior, lateral or paravertebral, posterior or spinal canal, venous, or multiple‐site leakage [17]. For multiple‐site leakage, each involved location was recorded. Disagreements were resolved by consensus, and the consensus imaging assessment served as the reference standard.

### 2.3 Segmentation and Preprocessing

Thoracic and lumbar spine CT scans were retrieved from the hospital picture archiving and communication system (PACS) and exported in DICOM format. CT images were acquired using different scanner manufacturers and models across participating centers. Detailed acquisition and reconstruction parameters, including tube voltage, tube current, slice thickness, pixel spacing, and reconstruction kernel, are summarized in Table S2. Images were resampled to isotropic voxel spacing (1.0 × 1.0 × 1.0 mm) and intensity‐normalized using *z*‐score normalization based on the global dataset mean and standard deviation to reduce inter‐scanner variability. Regions of interest (ROIs) were defined as the entire vertebral body, excluding posterior elements (pedicles, lamina, and spinous process) and adjacent intervertebral discs. ROI delineation was performed semi‐automatically using 3D Slicer (version 4.11), with manual correction. A representative example of ROI delineation is provided in Figure 1. Two physicians with more than 5 years of experience in musculoskeletal imaging independently performed ROI segmentation, and discrepancies were resolved by consensus. Interobserver agreement was assessed using the intraclass correlation coefficient (ICC), and features with ICC > 0.75 were retained (mean ICC = 0.87). The resulting ROI masks were saved in NIfTI format. Subsequently, the preprocessed CT images were cropped to the ROIs to facilitate downstream radiomics feature extraction and model development.

**FIGURE 1:**
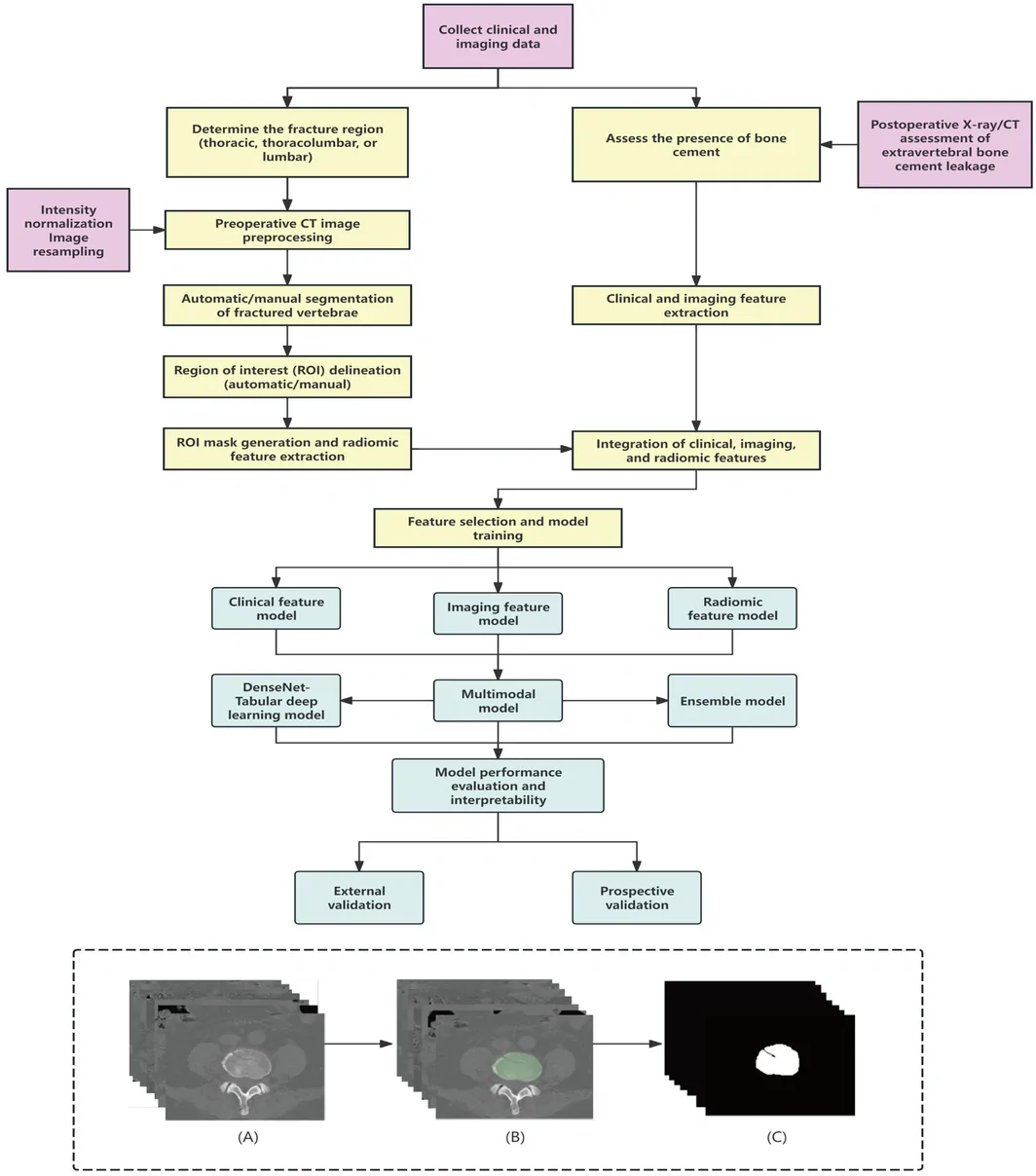
Overall workflow of data collection, image preprocessing, feature extraction, model development, and validation. The lower panels show a representative CT image (A), vertebral body ROI delineation (B), and the corresponding segmentation mask (C).

### 2.4 Clinical and Radiological Data

All variables were obtained from patients' medical records, imaging data (including preoperative MRI, pre‐ and postoperative CT scans, and anteroposterior and lateral spine radiographs), and surgical records.

### 2.5 Radiomics Features

High‐throughput radiomic features were extracted from semi‐automatically segmented vertebral ROIs on preoperative CT images using the Python‐based PyRadiomics package. In total, over 1700 features were generated, including first‐order statistical features, texture features (gray‐level co‐occurrence matrix [GLCM], gray‐level run‐length matrix [GLRLM], and gray‐level size zone matrix [GLSZM]), 3D shape features, and wavelet‐transformed features capturing multiscale characteristics.

### 2.6 Imaging Features

Conventional imaging features were independently evaluated on preoperative CT images by three spine surgeons with more than 5 years of experience in spinal imaging, focusing on established risk indicators for cement leakage. These included the presence of intravertebral cleft (IVC), cortical destruction, fracture type (wedge, biconcave, or crush) and severity (mild: 20%–25%, moderate: 26%–40%, severe: > 40% height loss), the basivertebral foramen sign, and posterior wall involvement defined as posterior wall protrusion into the spinal canal [8, 16, 18]. The surgeons were blinded to patients' clinical characteristics and the results of the statistical analyses. Interobserver agreement among the three surgeons was evaluated using the ICC, with a mean ICC of 0.84, indicating excellent agreement.

### 2.7 Clinical Features

Clinical variables were extracted from electronic medical records and included baseline characteristics (age, sex, body mass index [BMI], and BMD), medical history–related factors (corticosteroid use, anti‐osteoporotic medication, number and location of fractured vertebrae, comorbidities, and MRI‐based classification of fresh vs. old fractures), as well as surgical parameters (puncture approach and cement injection volume). The intended prediction window was perioperative, after procedural variables such as puncture approach and cement injection volume were available but before postoperative leakage assessment.

### 2.8 Model Development and Training

Data from the North, South, and Shenshan campuses formed the multicenter development cohort. Extracted features underwent missing‐value imputation and *z*‐score normalization. Radiomics features underwent univariate screening, near‐zero‐variance filtering, and correlation analysis, and were further selected using the least absolute shrinkage and selection operator (LASSO). All feature‐selection procedures were performed using the training data only, resulting in 15 radiomics features for the final comparative models. During nested cross‐validation, radiomics feature selection was repeated separately within each outer training fold.

The development cohort was divided at the patient level into training and internal test cohorts at an approximate 8:2 ratio. All vertebrae from the same patient were assigned to the same cohort and cross‐validation fold. Six machine‐learning algorithms—XGBoost, random forest (RF), decision tree (DT), Gaussian naïve Bayes (GNB), k‐nearest neighbors (KNN), and support vector machine (SVM)—were used to develop clinical, imaging, radiomics, and multimodal models. Model training, feature selection, and hyperparameter tuning were performed using the training cohort only, whereas the internal test cohort was used only for final model evaluation.

To evaluate the complete modeling workflow, all 24 algorithm–feature‐set combinations were further assessed using patient‐grouped nested cross‐validation with 10 outer folds and 5 inner folds. For models containing radiomics features, including the radiomics and multimodal models, variance filtering, correlation analysis, and LASSO feature selection were repeated using only the corresponding outer training fold. Hyperparameters for each of the six algorithms were determined using only the inner patient‐grouped cross‐validation folds. Each outer validation fold remained independent of feature selection and hyperparameter optimization. Performance was calculated from pooled predictions generated exclusively in the outer validation folds. The internal test cohort was not involved in the nested cross‐validation procedure. The patient‐grouped nested cross‐validation workflow is illustrated in Figure S1, and the predefined hyperparameter search spaces and complete implementation are provided in Code S1.

Multimodal fusion models were developed by integrating clinical, conventional imaging, and selected radiomics features using the six machine‐learning algorithms described above. An additional DenseNet‐inspired fully connected network (DenseNet‐Tabular) was evaluated as an exploratory supplementary model [19]. It was trained using binary cross‐entropy loss and evaluated using the same patient‐level data partitions.

### 2.9 Assessment of Site‐Related Radiomics Variability and Sensitivity Analysis

To assess potential site‐related variability in the 15 radiomics features retained for the final comparative models, additional analyses were performed using the training cohort only. Center‐related effects were evaluated using regression with cluster‐robust standard errors at the patient level and adjustment for leakage status, and false discovery rate (FDR) correction was performed using the Benjamini–Hochberg method. Center partial *R* ^2^ was used to quantify the magnitude of the adjusted center effect. Features showing concordant center effects before and after leakage adjustment with moderate‐to‐large adjusted effect sizes were considered site‐sensitive candidates. Three features (F06, F08, and F09) met these criteria and were excluded to construct a stable 12‐feature (Stable 12) sensitivity model. The six multimodal machine‐learning algorithms were retrained using the original patient‐level data split and patient‐grouped nested cross‐validation framework. The internal test, external validation, and prospective validation cohorts were not used for feature exclusion, model selection, or hyperparameter tuning.

### 2.10 Model Performance Evaluation and Interpretation

Model performance was assessed using AUC, accuracy, sensitivity (recall), specificity, positive predictive value (precision), negative predictive value, F1 score, and the Brier score. The classification threshold was determined using the training cohort and applied unchanged to the internal test, external validation, and prospective validation cohorts. Confidence intervals were calculated using patient‐cluster bootstrap resampling. Clinical utility was evaluated using decision curve analysis (DCA) across a range of threshold probabilities.

The multimodal algorithm with the highest pooled outer‐fold AUC in the training‐cohort nested cross‐validation was selected for model interpretation. SHapley Additive exPlanations (SHAP) were used to quantify and visualize feature contributions to model predictions [20]. Global SHAP analysis assessed the overall importance and direction of association of each feature, whereas local SHAP analysis illustrated feature contributions to individual predictions.

### 2.11 Ensemble Modeling

A comprehensive modeling framework was developed, including regularized models (LASSO, Ridge, ElasticNet), generalized linear models (Stepglm, glmBoost, PLS‐GLM), ensemble methods (Random Forest, GBM, XGBoost), and classical algorithms (SVM, LDA, Naive Bayes). The workflow followed two stages: feature subsets were generated using no feature selection (noFS), LASSO, Random Forest importance, Stepglm, or glmBoost; each subset was then processed using either the original features or PCA and trained using different algorithms, producing a total of 108 model configurations [21]. All model development and hyperparameter optimization were performed exclusively within the training cohort using patient‐grouped cross‐validation. After training was completed, each fitted configuration was applied without additional tuning to the internal test, external validation, and prospective validation cohorts. This separate robustness analysis assessed performance across different modeling strategies without using validation results to select or replace the final multimodal model. For each locked model configuration, the AUC and 95% confidence interval were calculated using 1000 patient‐cluster bootstrap resamples.

### 2.12 External and Prospective Validation

An independent external validation cohort comprising 50 treated vertebrae from 45 patients was obtained from another tertiary hospital. A prospective validation cohort comprising 60 treated vertebrae from 50 consecutive patients was enrolled at the North and South Campuses of our hospital between July and December 2025. Both cohorts followed the same inclusion and exclusion criteria as the development cohort. For both validations, feature alignment was performed, and the training‐derived preprocessing parameters and fitted multimodal models were applied unchanged without additional feature selection, retraining, or hyperparameter tuning. Performance was evaluated using AUC, accuracy, precision, recall, and F1 score, with 95% confidence intervals calculated using patient‐cluster bootstrap resampling. DCA was performed to assess clinical net benefit. The overall study workflow is illustrated in Figure 1, and the patient‐grouped nested cross‐validation workflow is shown in Figure S1.

### 2.13 Statistical Analysis

Data cleaning and statistical analyses were performed using Python (v3.6.5) and R (v4.4.1). Categorical variables were summarized as counts and percentages and compared using the Chi‐square or Fisher's exact test, as appropriate. Non‐normally distributed continuous variables were reported as medians with interquartile ranges (IQRs) and compared using the Mann–Whitney U test. The interobserver reliability of imaging assessments was evaluated using the ICC, categorized as poor (0–0.39), moderate (0.40–0.74), or excellent (0.75–1.00).

Patient‐level variables (e.g., age, sex, BMI, comorbidities) were assigned to all vertebrae from the same patient. To account for intra‐patient correlation arising from multiple vertebrae per patient, cluster‐adjusted analyses with robust standard errors at the patient level were performed. In the machine learning analyses, all vertebrae from the same patient were assigned to the same dataset partition and cross‐validation fold. As a sensitivity analysis, vertebral prediction probabilities were aggregated at the patient level using either the maximum or mean probability. For this analysis, a patient was considered leakage‐positive if leakage occurred in at least one treated vertebra. A separate prediction‐level sensitivity analysis was performed by randomly retaining one treated vertebra per patient in each of 200 iterations. Univariate logistic regression was used to identify variables associated with bone cement leakage. Variables with *p* < 0.05 were included in the multivariate logistic regression analysis, and the results were reported as odds ratios (ORs) with 95% confidence intervals (CIs).

Missing data were handled using multiple imputation by chained equations (MICE), which accommodates different variable types [22, 23]. The proportion of missing values and imputation diagnostics are provided in Table S3. Predictive modeling was primarily conducted using machine learning algorithms as described in the Model Development section. Performance estimates were reported with 95% confidence intervals obtained from 1000 patient‐cluster bootstrap resamples, except for the leakage‐location analyses, which used 2000 resamples. AUC differences between the Primary15 and Stable12 models were estimated using 1000 paired patient‐cluster bootstrap resamples. The 18 prespecified within‐algorithm comparisons between each multimodal model and its corresponding single‐modality models were evaluated using paired DeLong tests and 1000 paired patient‐cluster bootstrap resamples, with Bonferroni adjustment. All tests were two‐sided, with *p* < 0.05 considered statistically significant.

## 3 Results

### 3.1 Baseline Characteristics

The development cohort comprised 486 treated vertebrae from 378 patients, including 385 vertebrae from 301 patients in the training cohort and 101 vertebrae from 77 patients in the internal test cohort, with no patient overlap. Cement leakage occurred in 215 vertebrae (44.2%). The external and prospective validation cohorts included 50 vertebrae from 45 patients and 60 vertebrae from 50 patients, respectively. Patient‐ and vertebra‐level distributions are provided in Table S1, and baseline characteristics of the development cohort are summarized in Table 1. As shown in Table 1, the leakage group had higher cement injection volumes (3.50 vs. 2.00 mL), more frequent bipedicular puncture (74.4% vs. 58.3%), greater cortical destruction (85.6% vs. 21.8%), higher rates of posterior wall involvement (66.1% vs. 13.3%), and a higher prevalence of crush‐type or severe fractures. Higher BMI and a higher prevalence of hypertension were also observed in the leakage group. Characteristics of the external validation cohort are summarized in Table S4.

**TABLE 1:** Clinical and imaging characteristics and distribution of cement leakage patterns in the development cohort.

**Panel A**. Comparison of clinical and imaging variables by cement leakage status					
Variables		Non‐bone cement leakage (*n* = 271)	Bone cement leakage (*n* = 215)	Total (*n* = 486)	*p*
Baseline characteristics	Age (years)	72.00 (66.00, 80.00)	72.00 (65.50, 79.00)	72.00 (66.00, 79.00)	0.325
	Height (cm)	157.00 (150.00, 162.00)	156.00 (152.00, 160.00)	156.00 (151.00, 162.00)	0.688
	Weight (kg)	55.00 (49.50, 64.00)	56.00 (50.00, 65.00)	55.00 (50.00, 64.00)	0.059
	BMI (kg/m^2^)	22.22 (19.87, 25.33)	23.44 (20.66, 26.67)	22.60 (20.20, 25.97)	< 0.001*
	BMD	−3.20 (−3.90, −2.60)	−3.30 (−3.90, −2.50)	−3.30 (−3.90, −2.50)	0.879
	Sex				0.923
	Female	214 (78.97)	169 (78.60)	383 (78.81)	
	Male	57 (21.03)	46 (21.40)	103 (21.19)	
Medical history related variables	Hypertension				0.002*
	No	177 (65.31)	110 (51.16)	287 (59.05)	
	Yes	94 (34.69)	105 (48.84)	199 (40.95)	
	Diabetes				0.299
	No	229 (84.50)	174 (80.93)	403 (82.92)	
	Yes	42 (15.50)	41 (19.07)	83 (17.08)	
	Coronary heart disease				0.777
	No	249 (91.88)	196 (91.16)	445 (91.56)	
	Yes	22 (8.12)	19 (8.84)	41 (8.44)	
	Cerebrovascular disease				0.055
	No	253 (93.36)	190 (88.37)	443 (91.15)	
	Yes	18 (6.64)	25 (11.63)	43 (8.85)	
	History of steroid use				0.706
	No	255 (94.10)	204 (94.88)	459 (94.44)	
	Yes	16 (5.90)	11 (5.12)	27 (5.56)	
	History of anti‐osteoporosis drug use				0.562
	No	258 (95.20)	207 (96.28)	465 (95.68)	
	Yes	13 (4.80)	8 (3.72)	21 (4.32)	
	Number of vertebral fractures				0.350
	1	160 (59.04)	144 (66.98)	304 (62.55)	
	2	64 (23.62)	42 (19.53)	106 (21.81)	
	3	29 (10.70)	16 (7.44)	45 (9.26)	
	4	8 (2.95)	8 (3.72)	16 (3.29)	
	5	10 (3.69)	5 (2.33)	15 (3.09)	
	Fracture location				0.703
	Thoracic	91 (33.58)	80 (37.21)	171 (35.19)	
	Thoracolumbar	18 (6.64)	14 (6.51)	32 (6.58)	
	Lumbar	162 (59.78)	121 (56.28)	283 (58.23)	
Operation related variables	Puncture approach				< 0.001*
	Unipedicular	113 (41.70)	55 (25.58)	168 (34.57)	
	Bipedicular	158 (58.30)	160 (74.42)	318 (65.43)	
Radiology related variables	Volume of bone cement (mL)	2.00 (1.50, 3.00)	3.50 (3.00, 4.00)	3.00 (2.00, 3.50)	< 0.001*
	IVC				0.096
	No	230 (84.87)	170 (79.07)	400 (82.30)	
	Yes	41 (15.13)	45 (20.93)	86 (17.70)	
	Cortical destruction				< 0.001*
	No	212 (78.23)	31 (14.42)	243 (50.00)	
	Yes	59 (21.77)	184 (85.58)	243 (50.00)	
	Type of fracture				< 0.001*
	Wedge	180 (66.42)	124 (57.67)	304 (62.55)	
	Biconcave	56 (20.66)	35 (16.28)	91 (18.72)	
	Crush	35 (12.92)	56 (26.05)	91 (18.72)	
	Severity of fracture				< 0.001*
	Mild	168 (61.99)	97 (45.12)	265 (54.53)	
	Moderate	63 (23.25)	57 (26.51)	120 (24.69)	
	Severe	40 (14.76)	61 (28.37)	101 (20.78)	
	Basivertebral foramen sign				0.020*
	No	160 (59.04)	149 (69.30)	309 (63.58)	
	Yes	111 (40.96)	66 (30.70)	177 (36.42)	
	Posterior wall involvement				< 0.001*
	No	235 (86.72)	73 (33.95)	308 (63.37)	
	Yes	36 (13.28)	142 (66.05)	178 (36.63)	

Panel B. Mutually exclusive distribution of cement leakage patterns in the development cohort	
Leakage pattern	*n* (%)
No leakage	271 (55.8)
Intervertebral disc only	96 (19.8)
Anterior only	53 (10.9)
Lateral/paravertebral only	32 (6.6)
Posterior/spinal canal only	7 (1.4)
Venous only	7 (1.4)
Multiple‐site leakage	20 (4.1)

*Note:* **Panel A**: Statistical significance is denoted by an asterisk (*). **Panel B:** Percentages were calculated using all 486 treated vertebrae as the denominator. Leakage patterns were mutually exclusive. Bold values indicate statistical significance (*p* < 0.05).

Abbreviations: BMD, bone mineral density; BMI, body mass index; IVC, intravertebral cleft.

Logistic regression analysis with cluster‐adjusted standard errors (Table S5) showed that higher BMI, hypertension, bipedicular puncture, larger cement injection volume, cortical destruction, crush‐type/severe fractures, and posterior wall involvement were associated with cement leakage in univariate analysis. Multivariate analysis identified higher BMI (OR = 1.11), larger cement injection volume (OR = 6.18), cortical destruction (OR = 6.39), and posterior wall involvement (OR = 8.05) as independent risk factors, with cortical destruction and posterior wall involvement showing the strongest associations.

### 3.2 Model Development and Performance Comparison

Three single‐modality models (clinical, imaging, and radiomics) were developed using six machine‐learning algorithms: XGBoost, RF, DT, GNB, SVM, and KNN. After feature preprocessing and selection, each algorithm was trained separately within each feature set, and all six algorithms were retained and reported. The internal test cohort was used only for model evaluation and was not involved in feature selection or hyperparameter tuning.

The three single‐modality models showed distinct performance profiles in the internal test cohort. Clinical models demonstrated good discrimination, with DT achieving the highest AUC of 0.871. Imaging models also showed good discrimination, with GNB achieving the highest AUC of 0.844. Radiomics models showed moderate discrimination, with RF achieving the highest AUC of 0.806.

Clinical, conventional imaging, and selected radiomics features were then integrated to construct multimodal models. In the internal test cohort, the AUCs of the six multimodal algorithms ranged from 0.902 to 0.939, and the multimodal models consistently achieved a higher AUC than the corresponding single‐modality models within each algorithm. XGBoost showed the highest observed AUC among the multimodal models in the internal test cohort (AUC = 0.939; 95% CI, 0.886–0.977), followed by RF (AUC = 0.931) and DT (AUC = 0.918). ROC curves for all six algorithms in the training and test cohorts are shown in Figure 2. Within the internal test cohort, pairwise AUC comparisons between multimodal and corresponding single‐modality models were conducted within each algorithm using the DeLong test. After Bonferroni correction, multimodal models showed significantly higher AUCs in seven comparisons (all adjusted *p* < 0.05), five of which were also significant in patient‐cluster bootstrap analyses. No other comparisons remained significant after correction (Table S6).

**FIGURE 2:**
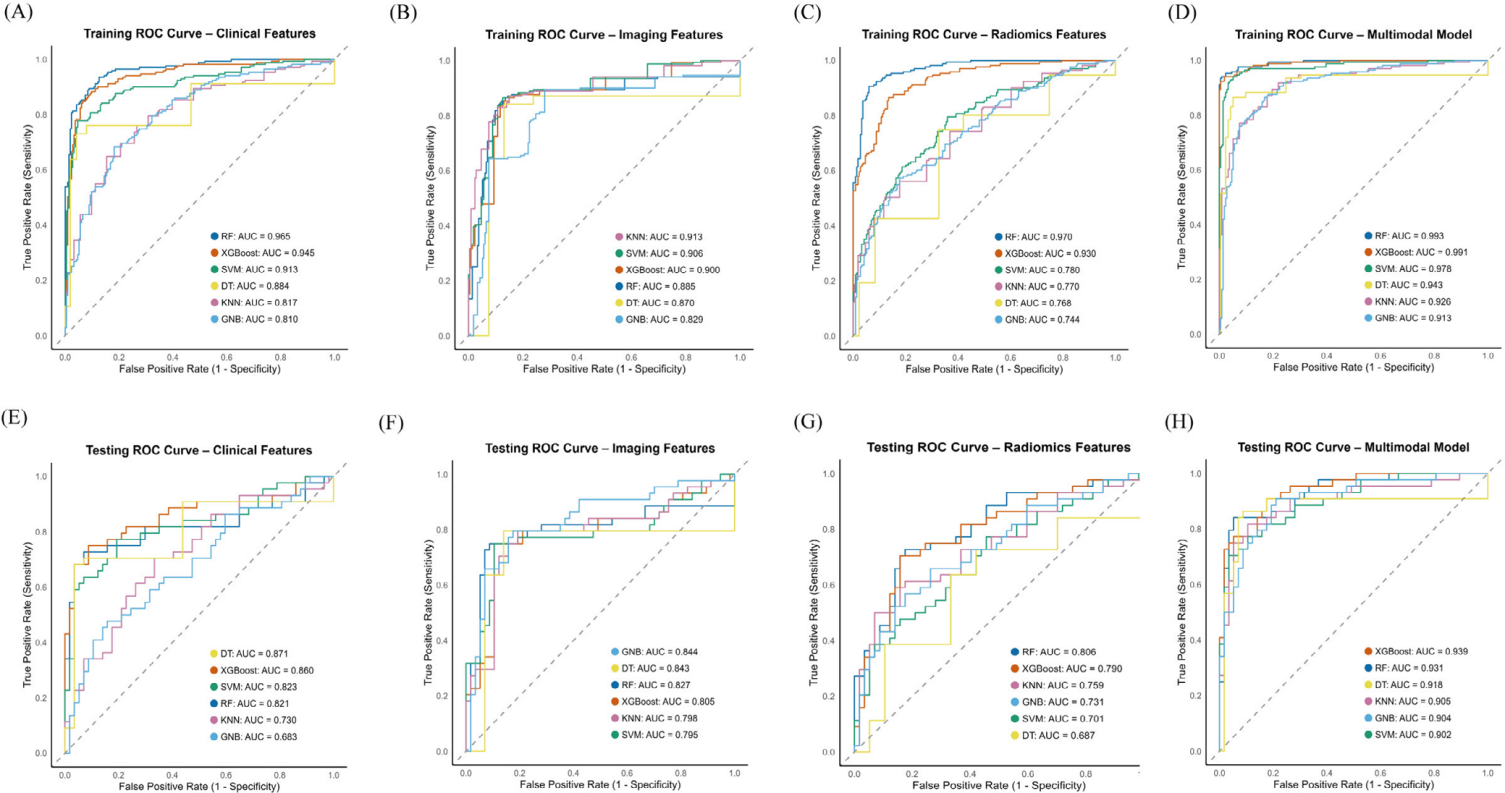
ROC curves of six machine‐learning algorithms across four feature sets. Panels A–D show the results in the training cohort for the clinical (A), imaging (B), radiomics (C), and multimodal (D) models. Panels E–H show the corresponding results in the internal test cohort for the clinical (E), imaging (F), radiomics (G), and multimodal (H) models. DT, decision tree; GNB, Gaussian naïve Bayes; KNN, k‐nearest neighbors; RF, random forest; SVM, support vector machine.

In the internal test cohort, DCA further showed that the multimodal models generally provided greater net benefit across clinically relevant threshold probabilities, particularly for RF and XGBoost (Figure 3A). Detailed performance metrics in the internal test cohort and corresponding patient‐cluster bootstrap 95% confidence intervals for all 24 models are provided in Table S7. The exploratory DenseNet‐based multimodal model achieved an AUC of 0.917, an accuracy of 87.1%, a precision of 0.897, and a recall of 79.5% in the internal test cohort (Figure S2).

**FIGURE 3:**
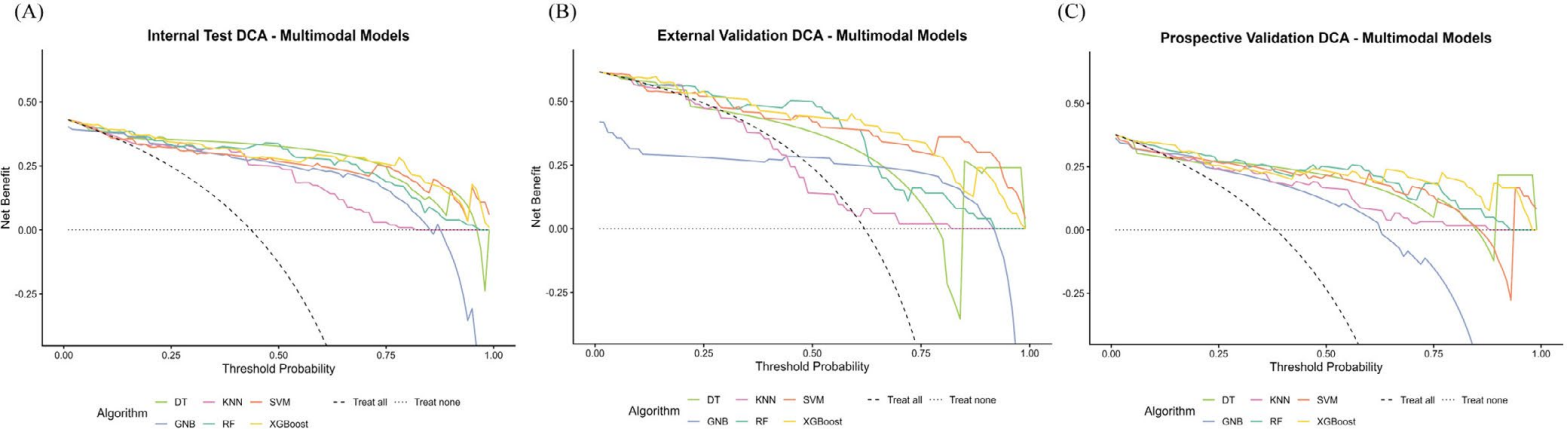
Decision curve analyses of the six multimodal models in the internal test cohort (A), external validation cohort (B), and prospective validation cohort (C). DT, decision tree; GNB, Gaussian naïve Bayes; KNN, k‐nearest neighbors; RF, random forest; SVM, support vector machine.

Patient‐level sensitivity analyses were performed for all six multimodal algorithms. After predictions from multiple treated vertebrae were aggregated at the patient level, the AUCs ranged from 0.883 to 0.912 using the maximum probability and from 0.865 to 0.895 using the mean probability. In 200 repeated analyses retaining one treated vertebra per patient, the mean AUCs ranged from 0.889 to 0.918 across the six algorithms. These results were generally consistent with the vertebral‐level findings, indicating that model performance was not substantially affected by the inclusion of multiple treated vertebrae from the same patient. Detailed results are provided in Table S8.

### 3.3 External and Prospective Validation of the Multimodal Models

In the external validation cohort (Figure 4A), most multimodal models demonstrated good generalizability, with AUCs ranging from 0.722 to 0.929. XGBoost showed the highest discriminative performance (AUC = 0.929; 95% CI, 0.837–0.988), followed closely by SVM (AUC = 0.927) and RF (AUC = 0.907). The corresponding classification metrics and DCA results are shown in Table S9 and Figure 3B, respectively.

**FIGURE 4:**
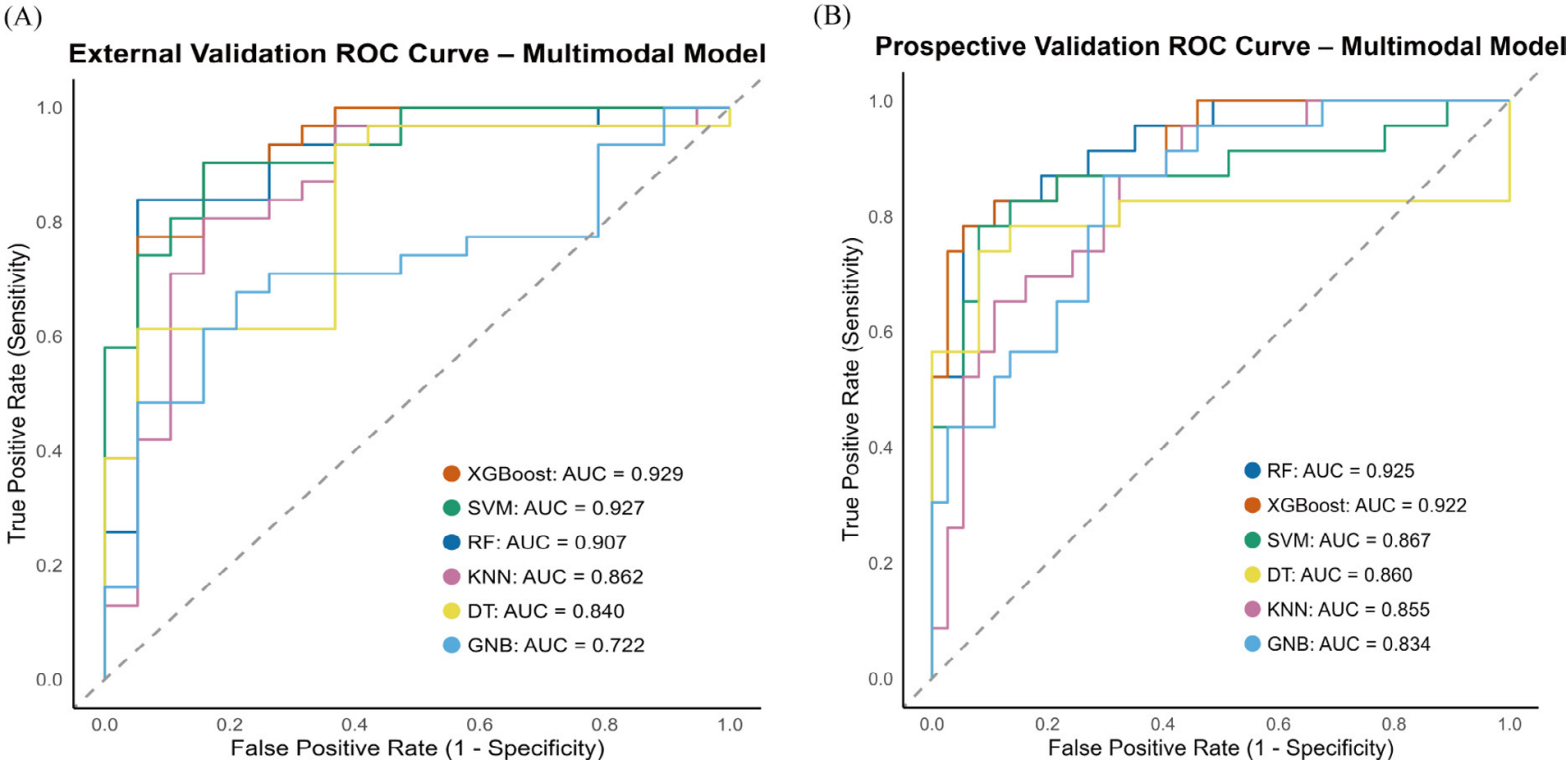
ROC curves of the six multimodal models in the external validation cohort (A) and prospective validation cohort (B).

In the prospective validation cohort (Figure 4B), the multimodal models maintained good predictive performance, with AUCs ranging from 0.834 to 0.925. RF achieved the highest discriminative performance (AUC = 0.925; 95% CI, 0.843–0.980), followed closely by XGBoost (AUC = 0.922), while SVM also showed good discrimination (AUC = 0.867). The corresponding classification metrics and DCA results are shown in Table S9 and Figure 3C, respectively.

The exploratory DenseNet‐based multimodal model achieved AUCs of 0.902 and 0.932 in the external and prospective validation cohorts, respectively, with corresponding accuracies of 82.0% and 86.7% (Figure S2). Overall, the multimodal models demonstrated good discriminative performance and generally stable classification across the external and prospective validation cohorts, supporting their potential clinical utility.

### 3.4 Model Explanation

Before the SHAP analysis, the six multimodal algorithms were compared using patient‐grouped nested cross‐validation in the training cohort. SVM achieved the highest pooled outer‐fold AUC of 0.936 (95% CI, 0.908–0.961), followed by RF (AUC = 0.924) and XGBoost (AUC = 0.917). Therefore, SVM was selected for SHAP‐based interpretation. The nested cross‐validation results are presented in Table S10.

Feature importance was assessed using the SHAP framework to provide both global and individual‐level interpretability. Global SHAP analysis highlighted cortical destruction, cement injection volume, and posterior wall involvement as the leading contributors to model predictions, with selected radiomics features providing additional complementary information (Figure 5A,B).

**FIGURE 5:**
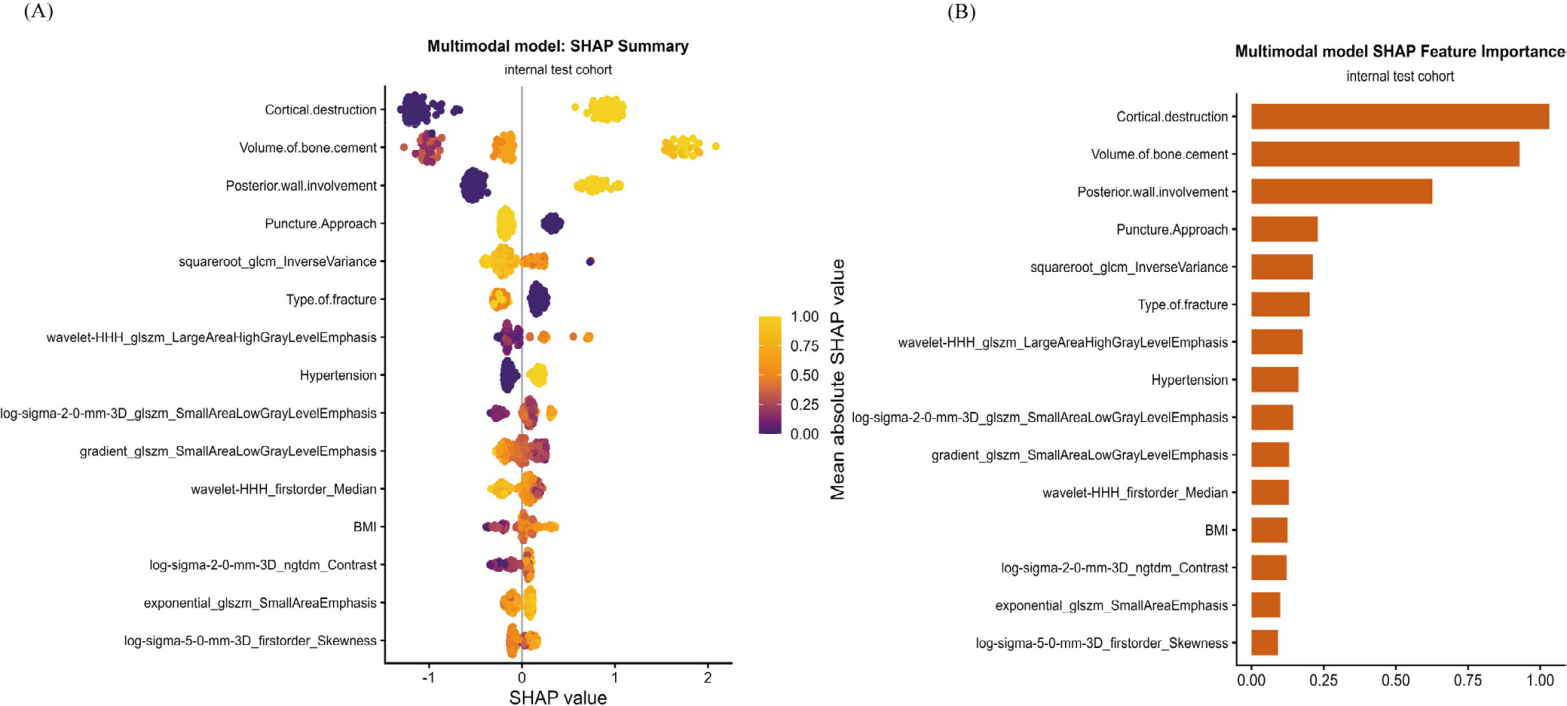
Global SHAP interpretation of the SVM multimodal model in the internal test cohort. (A) SHAP summary plot showing the distribution and direction of feature contributions to model predictions. (B) Ranking of the 15 most influential features according to their mean absolute SHAP values. SHAP, Shapley Additive Explanations; SVM, support vector machine.

Individual SHAP waterfall and force plots are shown in Figure S3. In representative cases, greater cement injection volume and the presence of cortical destruction or posterior wall involvement increased the predicted leakage risk, whereas lower cement injection volume and the absence of these findings reduced the predicted risk.

### 3.5 Leakage Location Distribution and Stratified Performance

Among the 215 leakage‐positive vertebrae in the development cohort, intervertebral disc involvement was observed in 108 (50.2%), anterior leakage in 69 (32.1%), lateral or paravertebral leakage in 40 (18.6%), posterior or spinal canal leakage in 11 (5.1%), and venous leakage in 7 (3.3%). Multiple‐site leakage occurred in 20 vertebrae (9.3%). Because multiple‐site cases were counted under each anatomical location involved, these percentages were not mutually exclusive. The mutually exclusive distribution of leakage patterns in the development cohort is presented in Table 1, with corresponding distributions across all cohorts provided in Table S11.

Using vertebrae without leakage as the reference group, the six multimodal algorithms showed good discrimination for the more common leakage patterns in the internal test cohort, with AUCs ranging from 0.850 to 0.904 for intervertebral disc leakage, from 0.918 to 0.967 for anterior leakage, and from 0.942 to 0.991 for lateral or paravertebral leakage. Performance remained generally favorable across the external and prospective cohorts, although some variation was observed across leakage types. Because posterior or spinal canal, venous, and multiple‐site leakage were uncommon, the corresponding results were interpreted descriptively. Venous leakage performance could not be estimated in the prospective cohort because no such event occurred. Detailed type‐specific results are provided in Table S12.

### 3.6 Site‐Related Radiomics Variability and Stable‐Feature Sensitivity Analysis

Among the 15 radiomics features retained for the final comparative models, three radiomics texture features (F06, F08, and F09) showed the strongest center‐related effects after adjustment for leakage status. Their adjusted center partial *R* ^2^ values were 0.092, 0.144, and 0.270, respectively. The selected first‐order features generally showed smaller center‐related effects than these three site‐sensitive transformed texture features. F06, F08, and F09 were therefore excluded to construct the Stable12 sensitivity model (Table S13, Panel A).

After exclusion of these features, model discrimination was broadly maintained across the six multimodal algorithms and the three validation cohorts. For SVM, the AUC changed from 0.902 to 0.866 in the internal test cohort, from 0.927 to 0.905 in the external validation cohort, and from 0.867 to 0.834 in the prospective validation cohort. The corresponding AUC differences were −0.036, −0.022, and −0.033, respectively. Similar patterns were observed for the other algorithms, with generally modest changes in AUC after exclusion of the three site‐sensitive features (Table S13, Panel B). These findings suggest that model performance was not primarily driven by a small number of center‐sensitive radiomics features.

### 3.7 Performance of Ensemble Models

A separate robustness analysis was performed using 108 locked model configurations. All configurations were developed within the training cohort, applied unchanged to the internal test, external validation, and prospective validation cohorts, and retained regardless of their validation performance. The median AUCs were 0.899 (IQR, 0.888–0.911), 0.919 (IQR, 0.893–0.935), and 0.860 (IQR, 0.835–0.874), respectively, with corresponding ranges of 0.850–0.946, 0.733–0.958, and 0.777–0.951 (Figure 6). These results showed generally consistent discrimination across different modeling configurations and validation cohorts. Configuration‐specific AUCs and patient‐cluster bootstrap 95% confidence intervals are provided in Data S1.

**FIGURE 6:**
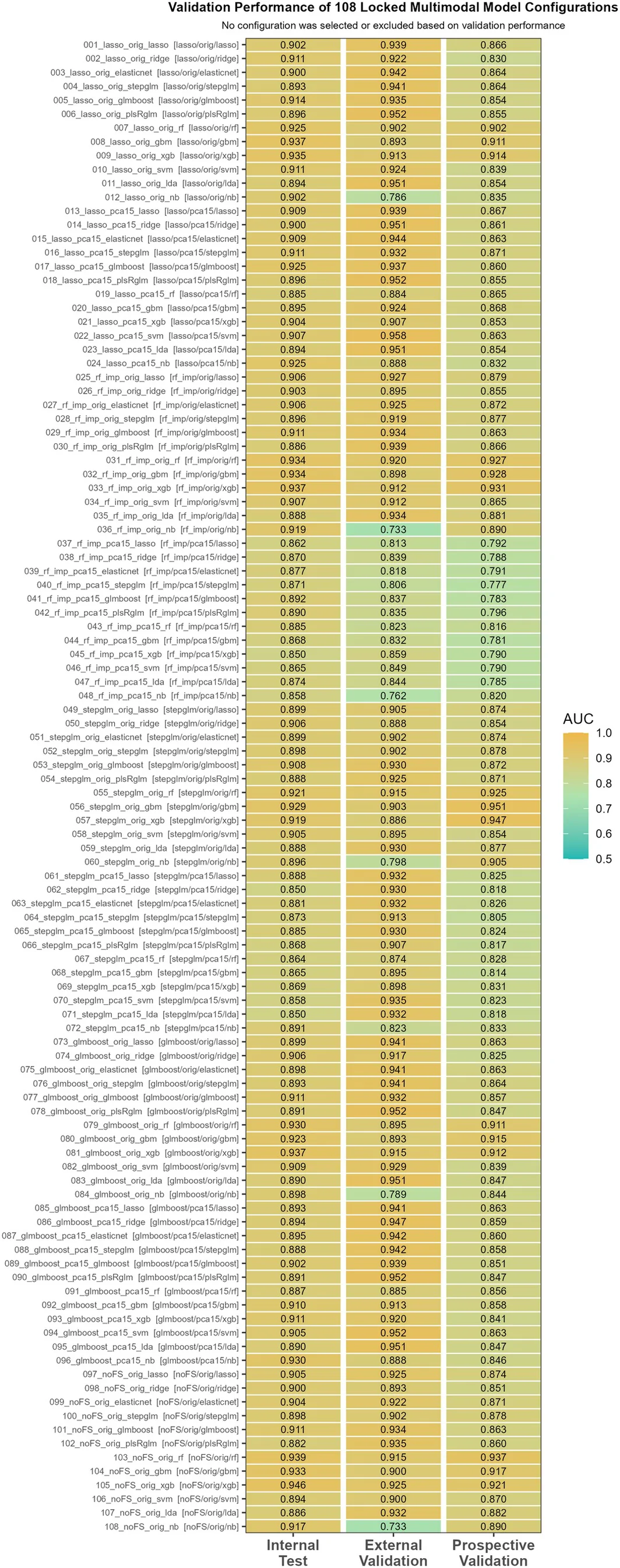
Heatmap of AUC values for 108 locked multimodal model configurations in the internal test, external validation, and prospective validation cohorts. Each row represents one model configuration, and each column represents one validation cohort. All configurations were retained, and none were selected or excluded according to their validation performance.

## 4 Discussion

This multicenter retrospective study developed a multimodal artificial intelligence model integrating clinical, imaging, and radiomics features to predict the risk of bone cement leakage during vertebroplasty. The development cohort included 486 treated vertebrae from 378 patients. Logistic regression analysis identified BMI, cement injection volume, and vertebral wall integrity (cortical destruction and posterior wall involvement) as independent risk factors for cement leakage. These findings are consistent with previous studies demonstrating the influence of cement volume and cortical defects on leakage risk, thereby further supporting their biological plausibility [24, 25, 26].

In recent years, ML and DL techniques have been increasingly applied to the diagnosis, treatment planning, and prognosis of spinal diseases. ML algorithms show high diagnostic accuracy with structured clinical and imaging data. For example, Zhang et al. combined multiple ML models with radiomics to differentiate benign from malignant vertebral compression fractures [27], while Chen et al. developed a CT‐based DL system for automatic vertebral segmentation and acute‐versus‐chronic fracture classification, achieving an AUC of 0.96 [28]. ML has also been applied to predict cement leakage risk after PVP. Li et al. established preoperative prediction models using multiple ML algorithms [15], and Deng et al. identified key predictors in 374 single‐level PVP patients and built multi‐algorithm models with favorable performance [29]. These studies support the application of ML and radiomics in cement leakage prediction. Our study further integrated clinical, conventional imaging, and radiomics features and evaluated the resulting models in both independent external and prospective cohorts. The differences in algorithm performance across cohorts also indicate the importance of comparing multiple algorithms rather than relying on a single modeling approach.

The multimodal models consistently achieved higher AUCs than the corresponding single‐modality models in the internal test cohort, reflecting the complementary value of clinical, imaging, and radiomic features. Compared with previous studies relying mainly on clinical or conventional imaging data, the incorporation of high‐dimensional radiomics may enable the models to capture subtle structural and textural heterogeneity, thereby improving predictive performance. Radiomics features should therefore be interpreted as preoperative imaging biomarkers associated with leakage susceptibility rather than causal determinants of leakage. Importantly, exclusion of the three radiomics features showing the strongest center‐related variability resulted in only modest changes in model discrimination across the validation cohorts. Together, these findings suggest that radiomics contributed complementary information to multimodal prediction, while the observed performance was not dependent on a small subset of center‐sensitive radiomics features. XGBoost achieved the highest observed AUCs in the internal test (AUC = 0.939) and external validation (AUC = 0.929) cohorts, whereas RF achieved the highest observed AUC in the prospective validation cohort (AUC = 0.925). These findings are consistent with previous evidence supporting the effectiveness of machine‐learning approaches for complex medical data analysis [30]. Unlike previous studies largely limited to internal test‐set evaluation, this study incorporated both independent external and prospective validation, further supporting the robustness, generalizability, and potential clinical applicability of the multimodal modeling framework.

A separate robustness analysis combining multiple feature‐selection, preprocessing, and learning strategies was performed to evaluate the stability of the multimodal modeling framework. The 108 configurations were developed using the training cohort and applied unchanged to the internal test, external validation, and prospective validation cohorts. The consistently good discrimination across these configurations further supported the robustness of the multimodal framework [21]. SHAP analysis provided global and individual‐level interpretability, highlighting cortical destruction, cement injection volume, and posterior wall involvement as important contributors to model predictions. Visualization of representative cases illustrated how the model integrates risk and protective factors for individualized predictions. Decision curve analysis demonstrated positive net benefit across clinically relevant threshold probabilities, with a generally favorable range of approximately 0.10–0.60 across the internal, external, and prospective cohorts. Within this range, a high predicted leakage risk may support more cautious cement injection, including slower injection, careful control of cement volume, use of higher‐viscosity cement when appropriate, and closer fluoroscopic monitoring. However, these potential risk‐mitigation strategies require prospective validation and should not be interpreted as prescriptive treatment rules.

This study has several limitations. First, CT images were acquired using different scanners and protocols. Three transformed texture features showed the strongest center‐related variability, although their exclusion resulted in only modest changes in model discrimination. A cross‐scanner ICC could not be calculated because repeated scans of the same participants or standardized phantoms across scanners were unavailable. Despite the center effect and stable feature analyses, residual acquisition‐related heterogeneity remains, and standardized imaging protocols or harmonization methods such as ComBat should be evaluated in future studies. Second, the primary outcome was radiographically detected cement leakage. Posterior or spinal canal and venous leakage were uncommon, and leakage volume, symptoms, neurological deficits, pulmonary embolism, and reoperation were not systematically recorded. Therefore, the models predict radiographic rather than symptomatic or clinically significant leakage. The small numbers of higher‐risk leakage patterns and the lack of detailed clinical outcomes precluded reliable development of a clinically meaningful multiclass model. Third, although patient‐grouped data partitioning, patient‐cluster bootstrap resampling, and patient‐level sensitivity analyses were applied, the treated vertebra remained the primary prediction unit, and residual within‐patient correlation cannot be completely excluded. Finally, despite independent external and prospective validation, further evaluation in larger and more diverse populations is required before wider clinical implementation.

## 5 Conclusions

This multicenter study developed a multimodal AI framework integrating clinical, imaging, and radiomics features to predict cement leakage during PVP. The multimodal models demonstrated consistent discrimination across the internal test, external validation, and prospective validation cohorts. SHAP analysis identified cortical destruction, cement injection volume, and posterior wall involvement as important contributors to model predictions. The framework may support perioperative risk stratification and individualized cement injection planning.

## Author Contributions

**Wangim Choi:** writing – original draft, formal analysis, methodology, conceptualization. **Jianan Chen:** writing – original draft, formal analysis, methodology, conceptualization. **Jincheng Qiu:** formal analysis, writing – original draft, validation. **Tianyu Xie:** validation, data curation. **Yong Li:** data curation. **Zhiwei Pan:** validation, data curation. **Zaoqiang Zhang:** investigation, data curation. **Guoxi Huang:** visualization, validation. **Nianchun Liao:** investigation, data curation. **Youxi Lin:** data curation, validation, funding acquisition. **Zhihuai Deng:** visualization, validation. **Xianjian Qiu:** validation, visualization, funding acquisition. **Bo Gao:** resources, project administration, funding acquisition. **Dongsheng Huang:** resources, funding acquisition, project administration. **Hang Zhou:** writing – review and editing, supervision, conceptualization. **Zizhao Wu:** writing – review and editing, conceptualization, supervision. **Wenjie Gao:** writing – review and editing, conceptualization, supervision.

## Funding

This study was supported by the National Natural Science Foundation of China (No. 82302737, No. 82372432, No. 82402183), Science and Technology Program of Guangzhou, China (No. 2024A04J4684, No. 2025A03J41881).

## Ethics Statement

This retrospective research had been approved by the Ethics Committee of Sun Yat‐sen Memorial Hospital (Ethical number SYSKY‐2025‐832‐01).

## Conflicts of Interest

The authors declare no conflicts of interest.

## Supporting information


**Figure S1:** Patient‐grouped nested cross‐validation and locked validation workflow. The development cohort was divided into training and internal test cohorts at the patient level. Within the training cohort, patient‐grouped nested cross‐validation with 10 outer folds and 5 inner folds was performed. Radiomics feature selection was repeated within each outer training fold, and hyperparameters were tuned using only the inner folds. After nested cross‐validation, the final models were refitted using the complete training cohort and applied without further feature selection, tuning, or modification to the internal test, external validation, and prospective validation cohorts.
**Figure S2:** Performance of the DenseNet‐based multimodal model. ROC curves and confusion matrices are shown for the internal test cohort (A, B), external validation cohort (C, D), and prospective validation cohort (E, F), respectively.
**Figure S3:** Individual SHAP explanations for four representative cases. Panels A and C show the waterfall and force plots for the first leakage case, whereas Panels B and D show the corresponding plots for the second leakage case. Panels E and G show the waterfall and force plots for the first non‐leakage case, whereas Panels F and H show the corresponding plots for the second non‐leakage case.
**Table S1:** Patient‐ and vertebra‐level distribution across study cohorts.
**Table S2:** CT scanner and acquisition parameters across study centers and validation cohorts.
**Table S3:** Missingness and imputation quality assessment for baseline variables.
**Table S4:** Comparison of clinical and imaging variables in the external validation cohort by cement leakage status.
**Table S5:** Univariate and multivariate logistic regression analysis of risk factors for bone cement leakage.
**Table S6:** Pairwise AUC comparisons between multimodal and corresponding single‐modality models in the internal test cohort.
**Table S7:** Performance of clinical, imaging, radiomics, and multimodal models in the internal test cohort.
**Table S8:** Patient‐level aggregation and one‐vertebra‐per‐patient sensitivity analyses of the six multimodal models.
**Table S9:** External and prospective validation performance of the multimodal models.
**Table S10:** Patient‐grouped nested cross‐validation performance of the six multimodal machine‐learning algorithms.
**Table S11:** Distribution of cement leakage patterns across study cohorts.
**Table S12:** Performance of the six multimodal algorithms stratified by cement leakage location.
**Table S13:** Site‐related variability of selected radiomics features and stable‐feature sensitivity analysis.
**Data S1:** AUCs and patient‐cluster bootstrap 95% CIs for 108 locked multimodal model configurations in the internal test, external validation, and prospective validation cohorts.
**Code S1**. R code for model development and validation using six machine‐learning algorithms, including patient‐grouped nested cross‐validation with 10 outer folds and 5 inner folds.

## Data Availability

The data that support the findings of this study are available from the corresponding author upon reasonable request.
