# Supplementary material for: Development of a Multimodal Model Combining Imaging and Clinical Data to Predict Bone Cement Leakage During Vertebroplasty for Fresh Thoracolumbar Compression Fractures
Source: JOR Spine. 2026 Nov 8;9(4):e70227. doi: 10.1002/jsp2.70227 (PMC13745800; doi:10.1002/jsp2.70227)
Supplement: Supplementary file 1 [file JSP2-9-e70227-s001.zip › Supplementary material/Supplementary Table S1.docx]

| **Table S1.** Patient- and vertebra-level distribution across study cohorts | | | | |
| --- | --- | --- | --- | --- |
| **Cohort** | **Patients, n** | **Treated vertebrae, n** | **Patients with 1 treated vertebra, n (%)** | **Patients with ≥2 treated vertebrae, n (%)** |
| Development cohort overall | 378 | 486 | 302 (79.9%) | 76 (20.1%) |
| Training cohort | 301 | 385 | 241 (80.1%) | 60 (19.9%) |
| Internal test cohort | 77 | 101 | 61 (79.2%) | 16 (20.8%) |
| External validation cohort | 45 | 50 | 40 (88.9%) | 5 (11.1%) |
| Prospective validation cohort | 50 | 60 | 41 (82.0%) | 9 (18.0%) |

**Note:** Percentages were calculated using the number of patients in each cohort as the denominator. All treated vertebrae from the same patient were assigned to the same dataset partition and cross-validation fold. No patient overlap was identified between the training and internal test cohorts or among the development, external validation, and prospective validation cohorts.
