# Supplementary material for: Development of a Multimodal Model Combining Imaging and Clinical Data to Predict Bone Cement Leakage During Vertebroplasty for Fresh Thoracolumbar Compression Fractures
Source: JOR Spine. 2026 Nov 8;9(4):e70227. doi: 10.1002/jsp2.70227 (PMC13745800; doi:10.1002/jsp2.70227)
Supplement: Supplementary file 1 [file JSP2-9-e70227-s001.zip › Supplementary material/Supplementary Table S10.docx]

| **Table S10.** Patient-grouped nested cross-validation performance of the six multimodal machine-learning algorithms | | | | | | | | |
| --- | --- | --- | --- | --- | --- | --- | --- | --- |
| **Algorithm** | **AUC (95% CI)** | **Accuracy (95% CI)** | **Sensitivity (95% CI)** | **Specificity (95% CI)** | **PPV (95% CI)** | **NPV (95% CI)** | **F1 score (95% CI)** | **Brier score (95% CI)** |
| SVM | 0.936 (0.908–0.961) | 0.883 (0.850–0.918) | 0.860 (0.802–0.913) | 0.902 (0.860–0.943) | 0.875 (0.820–0.926) | 0.889 (0.845–0.928) | 0.867 (0.823–0.907) | 0.092 (0.073–0.113) |
| RF | 0.924 (0.892–0.951) | 0.847 (0.807–0.881) | 0.807 (0.743–0.865) | 0.879 (0.833–0.921) | 0.841 (0.779–0.894) | 0.851 (0.800–0.900) | 0.824 (0.771–0.866) | 0.110 (0.094–0.128) |
| XGBoost | 0.917 (0.881–0.947) | 0.865 (0.830–0.900) | 0.842 (0.784–0.898) | 0.883 (0.837–0.928) | 0.852 (0.799–0.909) | 0.875 (0.831–0.920) | 0.847 (0.800–0.887) | 0.110 (0.091–0.130) |
| KNN | 0.901 (0.867–0.929) | 0.797 (0.757–0.836) | 0.632 (0.550–0.707) | 0.930 (0.893–0.961) | 0.878 (0.815–0.932) | 0.760 (0.702–0.810) | 0.735 (0.669–0.789) | 0.149 (0.136–0.164) |
| DT | 0.875 (0.830–0.914) | 0.839 (0.802–0.873) | 0.801 (0.739–0.858) | 0.869 (0.823–0.915) | 0.830 (0.767–0.891) | 0.845 (0.797–0.892) | 0.815 (0.764–0.858) | 0.126 (0.101–0.154) |
| GNB | 0.857 (0.820–0.896) | 0.792 (0.751–0.837) | 0.760 (0.695–0.831) | 0.818 (0.765–0.867) | 0.769 (0.708–0.831) | 0.810 (0.756–0.865) | 0.765 (0.709–0.819) | 0.176 (0.140–0.209) |

**Note:** Performance was evaluated using 10-outer/5-inner patient-grouped nested cross-validation in the training cohort (385 vertebrae from 301 patients). Values are point estimates (95% CIs), with 95% CIs calculated using 1,000 patient-cluster bootstrap resamples. Algorithms were ranked by pooled outer-fold AUC, and the internal test, external validation, and prospective validation cohorts were not used for model selection. SVM achieved the highest AUC and was selected for subsequent SHAP-based interpretation. DT = decision tree; GNB = Gaussian naive Bayes; KNN = k-nearest neighbors; NPV = negative predictive value; PPV = positive predictive value; RF = random forest; SVM = support vector machine.
