# Supplementary material for: Development of a Multimodal Model Combining Imaging and Clinical Data to Predict Bone Cement Leakage During Vertebroplasty for Fresh Thoracolumbar Compression Fractures
Source: JOR Spine. 2026 Nov 8;9(4):e70227. doi: 10.1002/jsp2.70227 (PMC13745800; doi:10.1002/jsp2.70227)
Supplement: Supplementary file 1 [file JSP2-9-e70227-s001.zip › Supplementary material/Supplementary Table S11.docx]

| **Table S11.** Distribution of cement leakage patterns across study cohorts | | | | | | | | | |
| --- | --- | --- | --- | --- | --- | --- | --- | --- | --- |
| **Cohort** | **Total vertebrae** | **No leakage, n (%)** | **Any leakage, n (%)** | **Intervertebral disc** | **Anterior** | **Lateral/paravertebral** | **Posterior/spinal canal** | **Venous** | **Multiple-site** |
| Development | 486 | 271 (55.8) | 215 (44.2) | 96 (19.8) | 53 (10.9) | 32 (6.6) | 7 (1.4) | 7 (1.4) | 20 (4.1) |
| Internal test | 101 | 57 (56.4) | 44 (43.6) | 18 (17.8) | 12 (11.9) | 8 (7.9) | 2 (2.0) | 1 (1.0) | 3 (3.0) |
| External validation | 50 | 19 (38.0) | 31 (62.0) | 6 (12.0) | 7 (14.0) | 11 (22.0) | 2 (4.0) | 1 (2.0) | 4 (8.0) |
| Prospective validation | 60 | 37 (61.7) | 23 (38.3) | 9 (15.0) | 8 (13.3) | 3 (5.0) | 1 (1.7) | 0 | 2 (3.3) |
| **Note:** Values are n (%), with percentages calculated using the total number of treated vertebrae in each cohort as the denominator. Leakage patterns were mutually exclusive and summed to the total number of vertebrae with any leakage. Multiple-site leakage was defined as involvement of more than one anatomical location. In the development cohort, the 20 multiple-site leakage cases comprised intervertebral disc + anterior (n=10), intervertebral disc + lateral/paravertebral (n=2), anterior + lateral/paravertebral (n=4), anterior + posterior/spinal canal (n=2), and lateral/paravertebral + posterior/spinal canal (n=2). | | | | | | | | | |
