# Supplementary material for: Development of a Multimodal Model Combining Imaging and Clinical Data to Predict Bone Cement Leakage During Vertebroplasty for Fresh Thoracolumbar Compression Fractures
Source: JOR Spine. 2026 Nov 8;9(4):e70227. doi: 10.1002/jsp2.70227 (PMC13745800; doi:10.1002/jsp2.70227)
Supplement: Supplementary file 1 [file JSP2-9-e70227-s001.zip › Supplementary material/Supplementary Table S12.docx]

| **Table S12.** Performance of the six multimodal algorithms stratified by cement leakage location | | | | | | |
| --- | --- | --- | --- | --- | --- | --- |
| **Internal test cohort** | | | | | | |
| **Algorithm** | **Intervertebral disc (positive n=20)** | **Anterior (positive n=15)** | **Lateral/paravertebral (positive n=8)** | **Posterior/spinal canal (positive n=3)** | **Venous (positive n=1)** | **Multiple-site leakage (positive n=3)** |
| **XGBoost** | 0.904 (0.811–0.969) | 0.953 (0.892–0.994) | 0.991 (0.966–1.000) | 0.994* | 1.000* | 0.994* |
| **RF** | 0.883 (0.777–0.967) | 0.967 (0.912–1.000) | 0.980 (0.942–1.000) | 0.991* | 0.982* | 0.994* |
| **DT** | 0.871 (0.761–0.961) | 0.941 (0.846–0.997) | 0.981 (0.947–1.000) | 0.991* | 0.991* | 0.991* |
| **GNB** | 0.850 (0.722–0.954) | 0.950 (0.892–0.989) | 0.945 (0.880–0.989) | 0.942* | 1.000* | 0.953* |
| **KNN** | 0.857 (0.695–0.967) | 0.942 (0.858–0.995) | 0.942 (0.859–0.995) | 0.977* | 1.000* | 0.971* |
| **SVM** | 0.864 (0.755–0.956) | 0.918 (0.807–0.993) | 0.956 (0.870–1.000) | 0.982* | 1.000* | 0.988* |

| **External validation cohort** | | | | | | |
| --- | --- | --- | --- | --- | --- | --- |
| **Algorithm** | **Intervertebral disc (positive n=9)** | **Anterior (positive n=9)** | **Lateral/paravertebral (positive n=12)** | **Posterior/spinal canal (positive n=4)** | **Venous (positive n=1)** | **Multiple-site leakage (positive n=4)** |
| **XGBoost** | 0.924 (0.807–1.000) | 0.906 (0.774–0.995) | 0.925 (0.825–0.995) | 0.961* | 1.000* | 0.908* |
| **RF** | 0.854 (0.639–1.000) | 0.912 (0.765–1.000) | 0.877 (0.714–1.000) | 0.961* | 0.947* | 0.776* |
| **DT** | 0.865 (0.702–0.988) | 0.716 (0.503–0.908) | 0.871 (0.720–0.975) | 0.895* | 0.974* | 0.803* |
| **GNB** | 0.713 (0.500–0.929) | 0.860 (0.648–1.000) | 0.614 (0.376–0.823) | 0.829* | 0.947* | 0.855* |
| **KNN** | 0.816 (0.567–0.990) | 0.895 (0.745–0.992) | 0.803 (0.601–0.955) | 0.934* | 0.921* | 0.743* |
| **SVM** | 0.883 (0.744–1.000) | 0.947 (0.850–1.000) | 0.908 (0.772–0.995) | 0.987* | 1.000* | 0.895* |

| **Prospective validation cohort** | | | | | | |
| --- | --- | --- | --- | --- | --- | --- |
| **Algorithm** | **Intervertebral disc (positive n=11)** | **Anterior (positive n=9)** | **Lateral/paravertebral (positive n=4)** | **Posterior/spinal canal (positive n=1)** | **Venous (positive n=0)** | **Multiple-site leakage (positive n=2)** |
| **XGBoost** | 0.948 (0.861–1.000) | 0.874 (0.711–0.992) | 0.980 (0.925–1.000) | 0.973* | NE* | 0.986* |
| **RF** | 0.956 (0.884–1.000) | 0.875 (0.733–0.987) | 0.959 (0.886–1.000) | 1.000* | NE* | 0.973* |
| **DT** | 0.921 (0.770–0.998) | 0.766 (0.540–0.982) | 0.926 (0.758–1.000) | 1.000* | NE* | 0.980* |
| **GNB** | 0.845 (0.713–0.952) | 0.844 (0.654–0.982) | 0.845 (0.641–1.000) | 0.892* | NE* | 0.986* |
| **KNN** | 0.886 (0.755–0.976) | 0.838 (0.655–0.965) | 0.845 (0.683–0.976) | 0.946* | NE* | 0.973* |
| **SVM** | 0.897 (0.697–0.998) | 0.817 (0.602–0.980) | 0.946 (0.859–1.000) | 0.946* | NE* | 1.000* |

**Note:** Values are AUC (95% CI). The number in each column header represents the number of positive vertebrae for the corresponding leakage location, rather than the total number of vertebrae included in that comparison. Vertebrae without cement leakage served as the reference group. For each location-specific analysis, the positive group included all vertebrae involving that anatomical location; therefore, multiple-site leakage cases contributed to each recorded component location. Vertebrae with leakage at other locations were excluded from the corresponding comparison. Confidence intervals for intervertebral-disc, anterior, and lateral/paravertebral leakage were calculated using 2,000 patient-cluster bootstrap resamples.* Descriptive point estimate only because posterior/spinal canal, venous, and multiple-site leakage were uncommon; confidence intervals were not estimated. NE, not estimable.
