# Supplementary material for: Development of a Multimodal Model Combining Imaging and Clinical Data to Predict Bone Cement Leakage During Vertebroplasty for Fresh Thoracolumbar Compression Fractures
Source: JOR Spine. 2026 Nov 8;9(4):e70227. doi: 10.1002/jsp2.70227 (PMC13745800; doi:10.1002/jsp2.70227)
Supplement: Supplementary file 1 [file JSP2-9-e70227-s001.zip › Supplementary material/Supplementary Table S13.docx]

| **Table S13.** Site-related variability of selected radiomics features and stable-feature sensitivity analysis | | | | | | | |
| --- | --- | --- | --- | --- | --- | --- | --- |
| **Panel A.** Site-related variability of the 15 selected radiomics features | | | | | | | |
| **Feature ID** | **Radiomics feature** | **Feature category** | **Unadjusted FDR** | **Unadjusted center R²** | **Leakage-adjusted FDR** | **Leakage-adjusted center partial R²** | **Interpretation** |
| F01 | log-sigma-2-0-mm-3D_glszm_SmallAreaLowGrayLevelEmphasis | LoG-GLSZM | <0.001 | 0.052 | <0.001 | 0.049 | Small residual center effect |
| F02 | log-sigma-2-0-mm-3D_gldm_DependenceVariance | LoG-GLDM | 0.108 | 0.018 | 0.449 | 0.007 | No strong evidence of center effect |
| F03 | log-sigma-2-0-mm-3D_ngtdm_Contrast | LoG-NGTDM | <0.001 | 0.055 | 0.016 | 0.027 | Small residual center effect |
| F04 | log-sigma-5-0-mm-3D_firstorder_Skewness | LoG-First-order | 0.004 | 0.025 | 0.024 | 0.016 | Small residual center effect |
| F05 | wavelet-HLL_glcm_MCC | Wavelet-GLCM | <0.001 | 0.067 | <0.001 | 0.054 | Center effect attenuated after Leakage adjustment |
| **F06** | **wavelet-HLH_glcm_Idn** | **Wavelet-GLCM** | **<0.001** | **0.084** | **<0.001** | **0.092** | **Site-sensitive candidate** |
| F07 | wavelet-HHH_firstorder_Median | Wavelet-First-order | 0.986 | <0.001 | 0.573 | 0.005 | No strong evidence of center effect |
| **F08** | **wavelet-HHH_glszm_LargeAreaHighGrayLevelEmphasis** | **Wavelet-GLSZM** | **<0.001** | **0.165** | **<0.001** | **0.144** | **Site-sensitive candidate** |
| **F09** | **squareroot_glcm_InverseVariance** | **Square-root GLCM** | **<0.001** | **0.286** | **<0.001** | **0.270** | **Site-sensitive candidate** |
| F10 | logarithm_glcm_Idn | Logarithm-GLCM | 0.005 | 0.043 | 0.070 | 0.024 | No strong evidence after Leakage adjustment |
| F11 | exponential_glszm_GrayLevelNonUniformity | Exponential-GLSZM | 0.351 | 0.011 | 0.833 | 0.001 | No strong evidence of center effect |
| F12 | exponential_glszm_LowGrayLevelZoneEmphasis | Exponential-GLSZM | 0.816 | 0.001 | 0.833 | 0.001 | No strong evidence of center effect |
| F13 | exponential_glszm_SmallAreaEmphasis | Exponential-GLSZM | 0.388 | 0.007 | 0.122 | 0.014 | No strong evidence of center effect |
| F14 | gradient_glszm_GrayLevelNonUniformity | Gradient-GLSZM | 0.001 | 0.049 | 0.107 | 0.018 | No strong evidence after Leakage adjustment |
| F15 | gradient_glszm_SmallAreaLowGrayLevelEmphasis | Gradient-GLSZM | <0.001 | 0.044 | 0.016 | 0.026 | Small residual center effect |

**Note for Panel A:** Site effects were assessed in the training cohort using patient-clustered robust regression with adjustment for leakage status. FDR was controlled using the Benjamini–Hochberg method. F06, F08, and F09 showed persistent center effects and were excluded in the stable-feature sensitivity analysis. FDR = false discovery rate; GLCM = gray-level co-occurrence matrix; GLDM = gray-level dependence matrix; GLSZM = gray-level size-zone matrix; LoG = Laplacian of Gaussian; NGTDM = neighboring gray-tone difference matrix.

| **Panel B.** Sensitivity analysis comparing the original 15-feature and stable 12-feature multimodal models | | | | | | | | | |
| --- | --- | --- | --- | --- | --- | --- | --- | --- | --- |
| **Algorithm** | **Internal test Primary15 AUC** | **Stable12 AUC** | **ΔAUC (95% CI)** | **External Primary15 AUC** | **Stable12 AUC** | **ΔAUC (95% CI)** | **Prospective Primary15 AUC** | **Stable12 AUC** | **ΔAUC (95% CI)** |
| DT | 0.918 | 0.877 | −0.041 (−0.105 to −0.002) | 0.840 | 0.887 | +0.048 (−0.043 to 0.132) | 0.860 | 0.858 | −0.002 (−0.017 to 0.009) |
| GNB | 0.904 | 0.891 | −0.013 (−0.029 to −0.001) | 0.722 | 0.735 | +0.014 (−0.012 to 0.050) | 0.834 | 0.832 | −0.002 (−0.022 to 0.015) |
| KNN | 0.905 | 0.894 | −0.011 (−0.032 to 0.006) | 0.862 | 0.815 | −0.047 (−0.103 to −0.003) | 0.855 | 0.864 | +0.009 (−0.027 to 0.047) |
| RF | 0.931 | 0.909 | −0.023 (−0.066 to 0.006) | 0.907 | 0.913 | +0.007 (−0.023 to 0.039) | 0.925 | 0.904 | −0.021 (−0.044 to −0.003) |
| **SVM** | **0.902** | **0.866** | **−0.036 (−0.084 to 0.002)** | **0.927** | **0.905** | **−0.022 (−0.093 to 0.036)** | **0.867** | **0.834** | **−0.033 (−0.072 to 0.000)** |
| XGBoost | 0.939 | 0.916 | −0.023 (−0.082 to 0.017) | 0.929 | 0.888 | −0.041 (−0.101 to 0.006) | 0.922 | 0.914 | −0.008 (−0.031 to 0.008) |

**Note for Panel B:** Primary15 denotes the original model using 15 radiomics features; Stable12 denotes the sensitivity model after excluding F06, F08, and F09. ΔAUC was calculated as Stable12 minus Primary15, with 95% CIs estimated using 1,000 paired patient-cluster bootstrap resamples. DT = decision tree; GNB = Gaussian naive Bayes; KNN = k-nearest neighbors; RF = random forest; SVM = support vector machine.
