# Supplementary material for: Development of a Multimodal Model Combining Imaging and Clinical Data to Predict Bone Cement Leakage During Vertebroplasty for Fresh Thoracolumbar Compression Fractures
Source: JOR Spine. 2026 Nov 8;9(4):e70227. doi: 10.1002/jsp2.70227 (PMC13745800; doi:10.1002/jsp2.70227)
Supplement: Supplementary file 1 [file JSP2-9-e70227-s001.zip › Supplementary material/Supplementary Table S2.docx]

| **Table S2.** CT scanner and acquisition parameters across study centers and validation cohorts | | | | | | | | |
| --- | --- | --- | --- | --- | --- | --- | --- | --- |
| **Center/Cohort** | **Manufacturer** | **Scanner model** | **Tube voltage, kVp** | **Tube current, mA** | **Slice thickness, mm** | **Actual slice spacing, mm** | **Pixel spacing, mm** | **Reconstruction kernel** |
| Development cohort—South | GE Medical Systems; UIH | Discovery CT750 HD; Revolution CT; Revolution EVO; uCT 780 | 120–140 | 193–499 | 1.00–1.25 | 1.00–1.25 | 0.293–0.883 | BONE; BONEPLUS; B_VSHARP_C; SOFT |
| Development cohort—North | GE Medical Systems; Siemens; UIH | Discovery CT750 HD; Revolution EVO; SOMATOM Force; uCT 530; uCT 780 | 90–150 | 56–451 | 1.00–1.25 | 0.70–1.00 | 0.204–0.977 | BONE; B_VSHARP_B; B_VSHARP_C; Br40s; Br59d; Br59s |
| Development cohort—Shenshan | GE Medical Systems; Siemens | Revolution CT; SOMATOM Drive | 100–140 | 103–683 | 0.625–1.25 | 0.625–1.25 | 0.236–0.721 | BONE; BONEPLUS; Bl57f; Br59s; Br60s |
| External validation cohort | Philips | Ingenuity CT; Ingenuity Flex | 120–140 | 134–475 | 1.00–2.00 | 0.70–1.00 | 0.154–0.432 | B; C |
| Prospective validation cohort | GE Medical Systems; Philips; Siemens; UIH | Revolution CT; Revolution EVO; SOMATOM Force; Spectral CT; uCT 780 | 110–140 | 113–499 | 1.00–1.25 | 1.00–1.25 | 0.176–0.699 | BONE; BONEPLUS; B_VSHARP_C; YA; Br59s |

**Note:** Continuous CT acquisition parameters are presented as observed ranges. Reconstruction kernels are reported using the original vendor-specific DICOM nomenclature. The tube-current–time product in mAs was not consistently available in the archived CT image DICOM metadata; therefore, tube current is reported in mA. kVp = peak kilovoltage; UIH = United Imaging Healthcare; mA = tube current.
