# Supplementary material for: Development of a Multimodal Model Combining Imaging and Clinical Data to Predict Bone Cement Leakage During Vertebroplasty for Fresh Thoracolumbar Compression Fractures
Source: JOR Spine. 2026 Nov 8;9(4):e70227. doi: 10.1002/jsp2.70227 (PMC13745800; doi:10.1002/jsp2.70227)
Supplement: Supplementary file 1 [file JSP2-9-e70227-s001.zip › Supplementary material/Supplementary Table S3.docx]

| **Table S3.** Missingness and Imputation Quality Assessment for Baseline Variables. | | | | | |
| --- | --- | --- | --- | --- | --- |
| Variable | Type | Missing Count | Missing Percent | RMSE | MSE |
| Height **(cm)** | Numeric | 78 | 16.05% | 3.434 | 11.795 |
| Weight **(kg)** | Numeric | 62 | 12.76% | 6.398 | 40.934 |
| BMD | Numeric | 76 | 15.64% | 0.712 | 0.507 |
| **Note:** BMD = bone mineral density. No missing values were observed for the remaining variables. The missing proportions for height, weight, and BMD ranged from 12% to 16% and were handled using multiple imputation by chained equations (MICE). Imputation performance for numeric variables was evaluated by randomly masking a subset of observed values and comparing imputed values with the corresponding true values using root mean square error (RMSE) and mean square error (MSE), with lower values indicating better accuracy. The imputation errors were acceptable, with RMSE values of 3.43 cm for height, 6.39 kg for weight, and 0.71 for BMD, indicating good agreement between imputed and observed data. | | | | | |
