# Supplementary material for: Development of a Multimodal Model Combining Imaging and Clinical Data to Predict Bone Cement Leakage During Vertebroplasty for Fresh Thoracolumbar Compression Fractures
Source: JOR Spine. 2026 Nov 8;9(4):e70227. doi: 10.1002/jsp2.70227 (PMC13745800; doi:10.1002/jsp2.70227)
Supplement: Supplementary file 1 [file JSP2-9-e70227-s001.zip › Supplementary material/Supplementary Table S4.docx]

### Table S4. Comparison of Clinical and Imaging Variables in the External Validation Cohort by Cement Leakage Status

| Variables |  | | Non-Bone cement leakage (n = 19) | Bone cement leakage(n = 31) | Total (n = 50) | *P value* |
| --- | --- | --- | --- | --- | --- | --- |
| **Baseline characteristics** | Age **(years)** | 74.00 (72.50, 80.00) | | 74.00 (70.50, 84.50) | 74.00 (71.00, 83.50) | 0.873 |
|  | Height **(cm)** | 160.00(152.00,164.00) | | 160.00(154.00,166.00) | 160.00(152.75,165.00) | 0.808 |
|  | Weight **(Kg)** | 60.00(48.00, 64.00) | | 55.00(50.50, 62.00) | 55.00(50.00, 64.00) | 0.575 |
|  | BMI **(Kg/m^2^)** | 22.94 (19.63, 26.87) | | 20.96 (19.69, 24.62) | 22.83 (19.66, 25.46) | 0.583 |
|  | BMD | -4.10 (-4.80, -3.65) | | -3.80 (-4.20, -3.30) | -3.95 (-4.52, -3.32) | 0.238 |
|  | Sex |  | |  |  | 0.997 |
|  | Female | 16 (84.21) | | 25 (80.65) | 41 (82.00) |  |
|  | Male | 3 (15.79) | | 6 (19.35) | 9 (18.00) |  |
| **Medical history related variables** | Hypertension |  | |  |  | 0.186 |
|  | No | 8 (42.11) | | 19 (61.29) | 27 (54.00) |  |
|  | Yes | 11 (57.89) | | 12 (38.71) | 23 (46.00) |  |
|  | Diabetes |  | |  |  | 0.952 |
|  | No | 15 (78.95) | | 26 (83.87) | 41 (82.00) |  |
|  | Yes | 4 (21.05) | | 5 (16.13) | 9 (18.00) |  |
|  | Coronary heart disease |  | |  |  | - |
|  | No | 19(100.00) | | 31 (100.00) | 50(100.00) |  |
|  | Yes | 0 (0.00) | | 0 (0.00) | 0(0.00) |  |
|  | Cerebrovascular disease |  | |  |  | 0.999 |
|  | No | 17 (89.47) | | 29 (93.55) | 46 (92.00) |  |
|  | Yes | 2 (10.53) | | 2 (6.45) | 4 (8.00) |  |
|  | History of steroid use |  | |  |  | - |
|  | No | 19(100.00) | | 31 (100.00) | 50(100.00) |  |
|  | Yes | 0 (0.00) | | 0 (0.00) | 0(0.00) |  |
|  | History of anti osteoporosis drug use |  | |  |  | 0.380 |
|  | No | 18 (94.74) | | 31 (100.00) | 49 (98.00) |  |
|  | Yes | 1 (5.26) | | 0 (0.00) | 1 (2.00) |  |
|  | Number of vertebral fractures |  | |  |  | 0.484 |
|  | 1 | 18 (94.74) | | 26 (83.87) | 44 (88.00) |  |
|  | 2 | 1 (5.26) | | 5 (16.13) | 6 (12.00) |  |
|  | Fracture location |  | |  |  | 0.196 |
|  | Thoracic | 8 (42.11) | | 6 (19.35) | 14 (28.00) |  |
|  | Thoracolumbar | 0 (0.00) | | 2 (6.45) | 2 (4.00) |  |
|  | Lumbar | 11 (57.89) | | 23 (74.19) | 34 (68.00) |  |
| **Operation related variables** | Puncture approach |  | |  |  | 0.991 |
|  | Unipedicular | 11 (57.89) | | 18 (58.06) | 29 (58.00) |  |
|  | Bipedicular | 8 (42.11) | | 13 (41.94) | 21 (42.00) |  |
|  | Volume of bone cement **(mL)** | 2.00 (1.50, 3.00) | | 2.00 (2.00, 4.00) | 2.00 (2.00, 3.75) | **0.018*** |
| **Radiology related variables** | IVC |  | |  |  | 0.610 |
|  | No | 14 (73.68) | | 26 (83.87) | 40 (80.00) |  |
|  | Yes | 5 (26.32) | | 5 (16.13) | 10 (20.00) |  |
|  | Cortical destruction | |  |  |  | **<0.001*** |
|  | No | | 18 (94.74) | 2 (6.45) | 20 (40.00) |  |
|  | Yes | | 1 (5.26) | 29 (93.55) | 30 (60.00) |  |
|  | Type of fracture | |  |  |  | 0.416 |
|  | Wedge | | 13 (68.42) | 15 (48.39) | 28 (56.00) |  |
|  | Biconcave | | 3 (15.79) | 7 (22.58) | 10 (20.00) |  |
|  | Crush | | 3 (15.79) | 9 (29.03) | 12 (24.00) |  |
|  | Severity of fracture | |  |  |  | **0.027*** |
|  | Mild | | 16 (84.21) | 15 (48.39) | 31 (62.00) |  |
|  | Moderate | | 3 (15.79) | 11 (35.48) | 14 (28.00) |  |
|  | Severe | | 0 (0.00) | 5 (16.13) | 5 (10.00) |  |
|  | Basivertebral foramen sign | |  |  |  | 0.740 |
|  | No | | 12 (63.16) | 21 (67.74) | 33 (66.00) |  |
|  | Yes | | 7 (36.84) | 10 (32.26) | 17 (34.00) |  |
|  | Posterior wall involvement | |  |  |  | **<0.001*** |
|  | No | | 18 (94.74) | 12 (38.71) | 30 (60.00) |  |
|  | Yes | | 1 (5.26) | 19 (61.29) | 20 (40.00) |  |

**Note:** BMI = Body mass index; BMD=Bone mineral density; IVC = Intravertebral cleft. Statistical significance is denoted by an asterisk (*)
