# Supplementary material for: Development of a Multimodal Model Combining Imaging and Clinical Data to Predict Bone Cement Leakage During Vertebroplasty for Fresh Thoracolumbar Compression Fractures
Source: JOR Spine. 2026 Nov 8;9(4):e70227. doi: 10.1002/jsp2.70227 (PMC13745800; doi:10.1002/jsp2.70227)
Supplement: Supplementary file 1 [file JSP2-9-e70227-s001.zip › Supplementary material/Supplementary Table S5.docx]

**Table S5.** Univariate and Multivariate Logistic Regression Analysis of Risk Factors for Bone Cement Leakage

| Variables | Univariate | |  | Multivariate | |
| --- | --- | --- | --- | --- | --- |
|  | OR (95%CI) | *P* |  | OR (95%CI) | *P* |
| Age **(years)** | 0.99 (0.97 ~ 1.01) | 0.244 |  |  |  |
| BMI **(Kg/m^2^)** | 1.10 (1.05 ~ 1.15) | **<0.001*** |  | 1.11 (1.03 ~ 1.20) | **0.007*** |
| BMD | 1.02 (0.86 ~ 1.20) | 0.766 |  |  |  |
| Sex |  |  |  |  |  |
| Female | 1.00 (Reference) |  |  |  |  |
| Male | 1.02 (0.66 ~ 1.58) | 0.923 |  |  |  |
| Hypertension |  |  |  |  |  |
| No | 1.00 (Reference) |  |  | 1.00 (Reference) |  |
| Yes | 1.80 (1.25 ~ 2.59) | **0.002*** |  | 1.37 (0.74 ~ 2.51) | 0.316 |
| Diabetes |  |  |  |  |  |
| No | 1.00 (Reference) |  |  |  |  |
| Yes | 1.28 (0.80 ~ 2.06) | 0.299 |  |  |  |
| Coronary heart disease |  |  |  |  |  |
| No | 1.00 (Reference) |  |  |  |  |
| Yes | 1.10 (0.58 ~ 2.08) | 0.777 |  |  |  |
| Cerebrovascular disease |  |  |  |  |  |
| No | 1.00 (Reference) |  |  |  |  |
| Yes | 1.85 (0.98 ~ 3.49) | 0.057 |  |  |  |
| History of steroid use |  |  |  |  |  |
| No | 1.00 (Reference) |  |  |  |  |
| Yes | 0.86 (0.39 ~ 1.89) | 0.707 |  |  |  |
| History of anti osteoporosis drug use |  |  |  |  |  |
| No | 1.00 (Reference) |  |  |  |  |
| Yes | 0.77 (0.31 ~ 1.89) | 0.563 |  |  |  |
| Number of vertebral fractures |  |  |  |  |  |
| 1 | 1.00 (Reference) |  |  |  |  |
| 2 | 0.73 (0.47 ~ 1.14) | 0.169 |  |  |  |
| 3 | 0.61 (0.32 ~ 1.17) | 0.140 |  |  |  |
| 4 | 1.11 (0.41 ~ 3.04) | 0.837 |  |  |  |
| 5 | 0.56 (0.19 ~ 1.66) | 0.294 |  |  |  |
| Fracture location |  |  |  |  |  |
| Thoracic | 1.00 (Reference) |  |  |  |  |
| Thoracolumbar | 0.88 (0.41 ~ 1.89) | 0.752 |  |  |  |
| Lumbar | 0.85 (0.58 ~ 1.24) | 0.403 |  |  |  |
| Puncture approach |  |  |  |  |  |
| Unipedicular | 1.00 (Reference) |  |  | 1.00 (Reference) |  |
| Bipedicular | 2.08 (1.41 ~ 3.07) | **<0.001*** |  | 0.09 (0.03 ~ 0.25) | **<0.001*** |
| Volume of bone cement **(mL)** | 11.26 (7.24 ~ 17.51) | **<0.001*** |  | 6.18 (4.45 ~ 8.60) | **<0.001*** |
| IVC |  |  |  |  |  |
| No | 1.00 (Reference) |  |  |  |  |
| Yes | 1.48 (0.93 ~ 2.37) | 0.097 |  |  |  |
| Cortical destruction |  |  |  |  |  |
| No | 1.00 (Reference) |  |  | 1.00 (Reference) |  |
| Yes | 13.46 (6.67 ~ 27.18) | **<0.001*** |  | 6.39 (3.07 ~ 13.28) | **<0.001*** |
| Type of fracture |  |  |  |  |  |
| Wedge | 1.00 (Reference) |  |  | 1.00 (Reference) |  |
| Biconcave | 0.91 (0.56 ~ 1.47) | 0.691 |  | 0.60 (0.27 ~ 1.31) | 0.200 |
| Crush | 2.32 (1.44 ~ 3.75) | **0.001*** |  | 0.89 (0.38 ~ 2.08) | 0.779 |
| Severity of fracture |  |  |  |  |  |
| Mild | 1.00 (Reference) |  |  | 1.00 (Reference) |  |
| Moderate | 1.54 (1.00 ~ 2.39) | 0.052 |  | 0.56 (0.22 ~ 1.38) | 0.206 |
| Severe | 2.49 (1.56 ~ 3.99) | **<0.001*** |  | 0.51 (0.18 ~ 1.44) | 0.205 |
| Basivertebral foramen sign |  |  |  |  |  |
| No | 1.00 (Reference) |  |  | 1.00 (Reference) |  |
| Yes | 0.66 (0.45 ~ 0.97) | **0.032*** |  | 0.85 (0.44 ~ 1.62) | 0.620 |
| Posterior wall involvement |  |  |  |  |  |
| No | 1.00 (Reference) |  |  | 1.00 (Reference) |  |
| Yes | 12.05 (7.71 ~ 18.83) | **<0.001*** |  | 8.05 (3.61 ~ 17.98) | **<0.001*** |

**Note:** BMI = Body mass index; BMD=Bone mineral density; IVC = Intravertebral cleft. Statistical significance is denoted by an asterisk (*)
