# Supplementary material for: Development of a Multimodal Model Combining Imaging and Clinical Data to Predict Bone Cement Leakage During Vertebroplasty for Fresh Thoracolumbar Compression Fractures
Source: JOR Spine. 2026 Nov 8;9(4):e70227. doi: 10.1002/jsp2.70227 (PMC13745800; doi:10.1002/jsp2.70227)
Supplement: Supplementary file 1 [file JSP2-9-e70227-s001.zip › Supplementary material/Supplementary Table S6.docx]

| **Table S6.** Pairwise AUC comparisons between multimodal and corresponding single-modality models in the internal test cohort | | | | | | | | | |
| --- | --- | --- | --- | --- | --- | --- | --- | --- | --- |
| **Algorithm** | **Comparison** | **Multimodal AUC** | **Single-modality AUC** | **ΔAUC** | **DeLong P** | **Bonferroni-adjusted DeLong P** | **Cluster-bootstrap ΔAUC (95% CI)** | **Cluster-bootstrap P** | **Bonferroni-adjusted bootstrap P** |
| RF | Multimodal vs Clinical | 0.931 | 0.821 | 0.111 | .015 | .261 | 0.111 (0.020–0.202) | .012 | .216 |
| RF | Multimodal vs Imaging | 0.931 | 0.827 | 0.104 | .004 | .069 | 0.104 (0.036–0.178) | .004 | .072 |
| RF | Multimodal vs Radiomics | 0.931 | 0.806 | 0.125 | .010 | .188 | 0.125 (0.027–0.222) | .020 | .360 |
| XGBoost | Multimodal vs Clinical | 0.939 | 0.860 | 0.079 | .048 | .858 | 0.079 (−0.005–0.171) | .068 | 1.000 |
| XGBoost | Multimodal vs Imaging | 0.939 | 0.805 | 0.134 | <.001 | .005 | 0.134 (0.058–0.208) | .002 | .036 |
| XGBoost | Multimodal vs Radiomics | 0.939 | 0.790 | 0.149 | .003 | .062 | 0.149 (0.052–0.249) | .010 | .180 |
| SVM | Multimodal vs Clinical | 0.902 | 0.823 | 0.079 | .009 | .158 | 0.079 (0.018–0.139) | .020 | .360 |
| SVM | Multimodal vs Imaging | 0.902 | 0.795 | 0.107 | .001 | .021 | 0.107 (0.043–0.183) | .002 | .036 |
| SVM | Multimodal vs Radiomics | 0.902 | 0.701 | 0.201 | .002 | .032 | 0.201 (0.076–0.315) | .006 | .108 |
| KNN | Multimodal vs Clinical | 0.905 | 0.730 | 0.175 | <.001 | .001 | 0.175 (0.102–0.248) | .002 | .036 |
| KNN | Multimodal vs Imaging | 0.905 | 0.798 | 0.107 | .011 | .199 | 0.107 (0.032–0.197) | .012 | .216 |
| KNN | Multimodal vs Radiomics | 0.905 | 0.759 | 0.146 | .019 | .344 | 0.146 (0.012–0.265) | .040 | .719 |
| DT | Multimodal vs Clinical | 0.918 | 0.871 | 0.047 | .228 | 1.000 | 0.047 (−0.025–0.115) | .220 | 1.000 |
| DT | Multimodal vs Imaging | 0.918 | 0.843 | 0.075 | .003 | .051 | 0.075 (0.023–0.133) | .004 | .072 |
| DT | Multimodal vs Radiomics | 0.918 | 0.687 | 0.230 | <.001 | .003 | 0.230 (0.100–0.375) | .004 | .072 |
| GNB | Multimodal vs Clinical | 0.904 | 0.683 | 0.220 | <.001 | <.001 | 0.220 (0.134–0.307) | .002 | .036 |
| GNB | Multimodal vs Imaging | 0.904 | 0.844 | 0.060 | .056 | 1.000 | 0.060 (0.009–0.122) | .030 | .539 |
| GNB | Multimodal vs Radiomics | 0.904 | 0.731 | 0.173 | <.001 | .009 | 0.173 (0.080–0.268) | .002 | .036 |

**Note:** ΔAUC represents the multimodal-model AUC minus the corresponding single-modality AUC. Paired DeLong tests and 1,000 paired patient-cluster bootstrap resamples were used. P values for the 18 prespecified comparisons were Bonferroni-adjusted; bootstrap 95% confidence intervals were unadjusted. Positive ΔAUC favors the multimodal model. DT = decision tree; GNB = Gaussian naive Bayes; KNN = k-nearest neighbors; RF = random forest; SVM = support vector machine.
