# Supplementary material for: Development of a Multimodal Model Combining Imaging and Clinical Data to Predict Bone Cement Leakage During Vertebroplasty for Fresh Thoracolumbar Compression Fractures
Source: JOR Spine. 2026 Nov 8;9(4):e70227. doi: 10.1002/jsp2.70227 (PMC13745800; doi:10.1002/jsp2.70227)
Supplement: Supplementary file 1 [file JSP2-9-e70227-s001.zip › Supplementary material/Supplementary Table S7.docx]

| **Table S7.** Performance of Clinical, Imaging, Radiomics, and Multimodal Models in the Internal Test Cohort | | | | | | | | | |
| --- | --- | --- | --- | --- | --- | --- | --- | --- | --- |
| **Model** | **Algorithm** | **AUC (95% CI)** | **Accuracy (95% CI)** | **Sensitivity (95% CI)** | **Specificity (95% CI)** | **PPV (95% CI)** | **NPV (95% CI)** | **F1-score (95% CI)** | **Brier score (95% CI)** |
| Clinical | XGBoost | 0.860 (0.765–0.943) | 0.842 (0.763–0.910) | 0.750 (0.610–0.875) | 0.912 (0.841–0.980) | 0.868 (0.757–0.970) | 0.825 (0.712–0.912) | 0.805 (0.687–0.889) | 0.134 (0.095–0.179) |
|  | RF | 0.821 (0.713–0.912) | 0.743 (0.649–0.837) | 0.773 (0.640–0.894) | 0.719 (0.578–0.862) | 0.680 (0.536–0.840) | 0.804 (0.696–0.913) | 0.723 (0.602–0.826) | 0.145 (0.093–0.201) |
|  | DT | 0.871 (0.807–0.926) | 0.842 (0.771–0.904) | 0.682 (0.543–0.811) | 0.965 (0.909–1.000) | 0.938 (0.844–1.000) | 0.797 (0.697–0.886) | 0.789 (0.677–0.875) | 0.124 (0.083–0.171) |
|  | GNB | 0.683 (0.583–0.778) | 0.653 (0.556–0.747) | 0.523 (0.378–0.673) | 0.754 (0.617–0.880) | 0.622 (0.444–0.794) | 0.672 (0.561–0.781) | 0.568 (0.429–0.695) | 0.251 (0.195–0.311) |
|  | SVM | 0.823 (0.722–0.908) | 0.772 (0.687–0.853) | 0.636 (0.488–0.778) | 0.877 (0.776–0.964) | 0.800 (0.636–0.938) | 0.758 (0.656–0.862) | 0.709 (0.575–0.814) | 0.158 (0.115–0.211) |
|  | KNN | 0.730 (0.646–0.819) | 0.683 (0.592–0.767) | 0.568 (0.420–0.714) | 0.772 (0.648–0.875) | 0.658 (0.500–0.806) | 0.698 (0.580–0.806) | 0.610 (0.475–0.725) | 0.210 (0.185–0.234) |
| Imaging | XGBoost | 0.805 (0.692–0.897) | 0.782 (0.684–0.865) | 0.750 (0.600–0.886) | 0.807 (0.691–0.899) | 0.750 (0.615–0.872) | 0.807 (0.673–0.913) | 0.750 (0.625–0.845) | 0.165 (0.111–0.231) |
|  | RF | 0.827 (0.710–0.920) | 0.782 (0.689–0.861) | 0.750 (0.595–0.878) | 0.807 (0.698–0.903) | 0.750 (0.609–0.867) | 0.807 (0.685–0.911) | 0.750 (0.621–0.854) | 0.166 (0.100–0.249) |
|  | DT | 0.843 (0.756–0.919) | 0.812 (0.723–0.890) | 0.750 (0.605–0.887) | 0.860 (0.766–0.941) | 0.805 (0.680–0.921) | 0.817 (0.697–0.922) | 0.776 (0.658–0.874) | 0.149 (0.094–0.213) |
|  | GNB | 0.844 (0.745–0.922) | 0.782 (0.691–0.866) | 0.795 (0.660–0.923) | 0.772 (0.653–0.875) | 0.729 (0.588–0.850) | 0.830 (0.705–0.930) | 0.761 (0.650–0.860) | 0.171 (0.103–0.247) |
|  | SVM | 0.795 (0.689–0.891) | 0.802 (0.719–0.876) | 0.750 (0.605–0.878) | 0.842 (0.745–0.925) | 0.786 (0.658–0.898) | 0.814 (0.696–0.911) | 0.767 (0.650–0.857) | 0.155 (0.100–0.219) |
|  | KNN | 0.798 (0.691–0.892) | 0.792 (0.708–0.875) | 0.750 (0.605–0.878) | 0.825 (0.723–0.915) | 0.767 (0.634–0.886) | 0.810 (0.696–0.912) | 0.759 (0.644–0.857) | 0.171 (0.112–0.232) |
| Radiomics | XGBoost | 0.790 (0.704–0.879) | 0.752 (0.664–0.839) | 0.750 (0.622–0.865) | 0.754 (0.625–0.881) | 0.702 (0.558–0.841) | 0.796 (0.679–0.900) | 0.725 (0.621–0.815) | 0.225 (0.216–0.235) |
|  | RF | 0.806 (0.721–0.888) | 0.743 (0.657–0.832) | 0.727 (0.600–0.850) | 0.754 (0.632–0.868) | 0.696 (0.571–0.837) | 0.782 (0.667–0.889) | 0.711 (0.602–0.817) | 0.184 (0.155–0.217) |
|  | DT | 0.687 (0.564–0.804) | 0.653 (0.543–0.757) | 0.636 (0.489–0.781) | 0.667 (0.522–0.792) | 0.596 (0.446–0.744) | 0.704 (0.564–0.828) | 0.615 (0.483–0.733) | 0.223 (0.171–0.273) |
|  | GNB | 0.731 (0.643–0.812) | 0.693 (0.612–0.773) | 0.568 (0.429–0.706) | 0.789 (0.684–0.878) | 0.676 (0.533–0.810) | 0.703 (0.583–0.809) | 0.617 (0.500–0.722) | 0.214 (0.168–0.265) |
|  | SVM | 0.701 (0.605–0.801) | 0.634 (0.550–0.723) | 0.727 (0.595–0.857) | 0.561 (0.444–0.680) | 0.561 (0.441–0.685) | 0.727 (0.596–0.860) | 0.634 (0.526–0.732) | 0.214 (0.183–0.244) |
|  | KNN | 0.759 (0.665–0.846) | 0.733 (0.640–0.816) | 0.614 (0.476–0.762) | 0.825 (0.714–0.914) | 0.730 (0.588–0.857) | 0.734 (0.622–0.839) | 0.667 (0.541–0.776) | 0.209 (0.185–0.233) |
| Multimodal | XGBoost | 0.939 (0.886–0.977) | 0.842 (0.758–0.907) | 0.773 (0.629–0.897) | 0.895 (0.812–0.965) | 0.850 (0.730–0.949) | 0.836 (0.724–0.926) | 0.810 (0.701–0.894) | 0.101 (0.064–0.149) |
|  | RF | 0.931 (0.874–0.978) | 0.891 (0.817–0.953) | 0.841 (0.707–0.947) | 0.930 (0.855–0.985) | 0.902 (0.795–0.980) | 0.883 (0.778–0.967) | 0.871 (0.776–0.946) | 0.107 (0.072–0.149) |
|  | DT | 0.918 (0.854–0.969) | 0.891 (0.825–0.948) | 0.841 (0.725–0.941) | 0.930 (0.857–0.985) | 0.902 (0.800–0.978) | 0.883 (0.796–0.955) | 0.871 (0.780–0.938) | 0.096 (0.048–0.150) |
|  | GNB | 0.904 (0.822–0.962) | 0.812 (0.722–0.891) | 0.795 (0.666–0.917) | 0.825 (0.714–0.917) | 0.778 (0.639–0.897) | 0.839 (0.719–0.939) | 0.787 (0.676–0.879) | 0.138 (0.076–0.211) |
|  | SVM | 0.902 (0.827–0.959) | 0.832 (0.745–0.904) | 0.773 (0.619–0.895) | 0.877 (0.789–0.958) | 0.829 (0.703–0.936) | 0.833 (0.719–0.925) | 0.800 (0.690–0.882) | 0.124 (0.074–0.188) |
|  | KNN | 0.905 (0.812–0.970) | 0.822 (0.734–0.899) | 0.818 (0.683–0.931) | 0.825 (0.717–0.918) | 0.783 (0.658–0.896) | 0.855 (0.740–0.950) | 0.800 (0.692–0.889) | 0.152 (0.122–0.190) |

**Note:** Data are presented as point estimates with 95% confidence intervals derived from 1,000 patient-cluster bootstrap resamples. Models were evaluated in the internal test cohort without additional feature selection or hyperparameter tuning. AUC indicates area under the receiver operating characteristic curve; PPV, positive predictive value; NPV, negative predictive value.
