# Supplementary material for: Development of a Multimodal Model Combining Imaging and Clinical Data to Predict Bone Cement Leakage During Vertebroplasty for Fresh Thoracolumbar Compression Fractures
Source: JOR Spine. 2026 Nov 8;9(4):e70227. doi: 10.1002/jsp2.70227 (PMC13745800; doi:10.1002/jsp2.70227)
Supplement: Supplementary file 1 [file JSP2-9-e70227-s001.zip › Supplementary material/Supplementary Table S8.docx]

| **Table S8.** Patient-level aggregation and one-vertebra-per-patient sensitivity analyses of the six multimodal models | | | | | |
| --- | --- | --- | --- | --- | --- |
| **Algorithm** | **Vertebral-level AUC** | **Patient-level AUC, maximum probability** | **Patient-level AUC, mean probability** | **Mean AUC from 200 iterations** | **2.5th–97.5th percentile range** |
| RF | 0.931 | 0.912 | 0.895 | 0.916 | 0.909–0.922 |
| XGBoost | 0.939 | 0.910 | 0.895 | 0.918 | 0.910–0.925 |
| SVM | 0.902 | 0.883 | 0.877 | 0.889 | 0.875–0.902 |
| KNN | 0.905 | 0.888 | 0.870 | 0.894 | 0.886–0.902 |
| DT | 0.918 | 0.899 | 0.889 | 0.897 | 0.891–0.901 |
| GNB | 0.904 | 0.890 | 0.865 | 0.890 | 0.870–0.907 |

**Note:** Vertebral-level AUCs were calculated using all treated vertebrae in the internal test cohort. For patient-level analyses, predictions from multiple treated vertebrae in the same patient were aggregated using either the maximum or mean predicted probability. In the one-vertebra-per-patient sensitivity analysis, one treated vertebra was randomly selected from each patient in 200 independent iterations; the mean AUC and 2.5th–97.5th percentile range across iterations are reported. DT = decision tree; GNB = Gaussian naive Bayes; KNN = k-nearest neighbors; RF = random forest; SVM = support vector machine.
