# Supplementary material for: Development of a Multimodal Model Combining Imaging and Clinical Data to Predict Bone Cement Leakage During Vertebroplasty for Fresh Thoracolumbar Compression Fractures
Source: JOR Spine. 2026 Nov 8;9(4):e70227. doi: 10.1002/jsp2.70227 (PMC13745800; doi:10.1002/jsp2.70227)
Supplement: Supplementary file 1 [file JSP2-9-e70227-s001.zip › Supplementary material/Supplementary Table S9.docx]

| **Table S9.** External and Prospective Validation Performance of the Multimodal Models | | | | | | | | |
| --- | --- | --- | --- | --- | --- | --- | --- | --- |
| **Panel A.** External validation cohort | | | | | | | | |
| **Algorithm** | **AUC (95% CI)** | **Accuracy (95% CI)** | **Sensitivity (95% CI)** | **Specificity (95% CI)** | **PPV (95% CI)** | **NPV (95% CI)** | **F1-score (95% CI)** | **Brier score (95% CI)** |
| XGBoost | 0.929 (0.837–0.988) | 0.820 (0.714–0.917) | 0.774 (0.621–0.906) | 0.895 (0.737–1.000) | 0.923 (0.808–1.000) | 0.708 (0.524–0.880) | 0.842 (0.720–0.925) | 0.123 (0.071–0.183) |
| RF | 0.907 (0.800–0.982) | 0.880 (0.792–0.959) | 0.839 (0.714–0.957) | 0.947 (0.818–1.000) | 0.963 (0.870–1.000) | 0.783 (0.600–0.944) | 0.897 (0.808–0.967) | 0.144 (0.100–0.195) |
| DT | 0.840 (0.726–0.948) | 0.760 (0.642–0.880) | 0.839 (0.714–0.960) | 0.632 (0.391–0.875) | 0.788 (0.622–0.938) | 0.706 (0.467–0.917) | 0.812 (0.698–0.907) | 0.179 (0.095–0.263) |
| GNB | 0.722 (0.572–0.856) | 0.640 (0.500–0.769) | 0.484 (0.312–0.667) | 0.895 (0.722–1.000) | 0.882 (0.700–1.000) | 0.515 (0.333–0.700) | 0.625 (0.450–0.764) | 0.339 (0.215–0.472) |
| SVM | 0.927 (0.849–0.985) | 0.800 (0.673–0.915) | 0.742 (0.560–0.900) | 0.895 (0.750–1.000) | 0.920 (0.783–1.000) | 0.680 (0.481–0.870) | 0.821 (0.679–0.926) | 0.138 (0.072–0.211) |
| KNN | 0.862 (0.734–0.964) | 0.780 (0.673–0.885) | 0.710 (0.562–0.864) | 0.895 (0.737–1.000) | 0.917 (0.793–1.000) | 0.654 (0.480–0.839) | 0.800 (0.691–0.899) | 0.243 (0.184–0.302) |

| **Panel B.** Prospective validation cohort | | | | | | | | |
| --- | --- | --- | --- | --- | --- | --- | --- | --- |
| **Algorithm** | **AUC (95% CI)** | **Accuracy (95% CI)** | **Sensitivity (95% CI)** | **Specificity (95% CI)** | **PPV (95% CI)** | **NPV (95% CI)** | **F1-score (95% CI)** | **Brier score (95% CI)** |
| XGBoost | 0.922 (0.834–0.978) | 0.850 (0.746–0.933) | 0.826 (0.640–0.958) | 0.865 (0.742–0.971) | 0.792 (0.609–0.950) | 0.889 (0.775–0.974) | 0.809 (0.649–0.921) | 0.116 (0.072–0.172) |
| RF | 0.925 (0.843–0.980) | 0.850 (0.750–0.936) | 0.826 (0.666–0.960) | 0.865 (0.744–0.972) | 0.792 (0.608–0.952) | 0.889 (0.780–0.974) | 0.809 (0.667–0.920) | 0.116 (0.082–0.155) |
| DT | 0.860 (0.748–0.956) | 0.833 (0.725–0.926) | 0.783 (0.615–0.944) | 0.865 (0.733–0.975) | 0.783 (0.594–0.960) | 0.865 (0.757–0.964) | 0.783 (0.615–0.906) | 0.138 (0.067–0.215) |
| GNB | 0.834 (0.724–0.924) | 0.733 (0.596–0.855) | 0.783 (0.615–0.947) | 0.703 (0.529–0.853) | 0.621 (0.423–0.815) | 0.839 (0.710–0.964) | 0.692 (0.522–0.840) | 0.220 (0.129–0.327) |
| SVM | 0.867 (0.734–0.967) | 0.800 (0.683–0.902) | 0.870 (0.706–1.000) | 0.757 (0.606–0.903) | 0.690 (0.486–0.875) | 0.903 (0.786–1.000) | 0.769 (0.600–0.892) | 0.137 (0.075–0.206) |
| KNN | 0.855 (0.745–0.942) | 0.717 (0.596–0.828) | 0.913 (0.792–1.000) | 0.595 (0.441–0.750) | 0.583 (0.412–0.743) | 0.917 (0.778–1.000) | 0.712 (0.554–0.833) | 0.164 (0.129–0.198) |

**Note:** Data are presented as point estimates with patient-cluster bootstrap 95% confidence intervals in parentheses. Multimodal models developed in the training cohort were applied unchanged to the external and prospective validation cohorts without additional training or hyperparameter tuning.
